# First-in-human phase I/Ib study of QL1706 (PSB205), a bifunctional PD1/CTLA4 dual blocker, in patients with advanced solid tumors

**DOI:** 10.1186/s13045-023-01445-1

**Published:** 2023-05-08

**Authors:** Yuanyuan Zhao, Yuxiang Ma, Aimin Zang, Ying Cheng, Yiping Zhang, Xiangcai Wang, Zhendong Chen, Song Qu, Jianbo He, Chuanben Chen, Chuan Jin, Dongyuan Zhu, Qingshan Li, Xianling Liu, Wuyun Su, Yi Ba, Yanrong Hao, Junmin Chen, Guoping Zhang, Shenhong Qu, Yong Li, Weineng Feng, Mengxiang Yang, Baorui Liu, Weiwei Ouyang, Jin Liang, Zhuang Yu, Xiaoyan Kang, Shilin Xue, Guihong Yang, Wei Yan, Yingying Yang, Zhi Liu, Yufeng Peng, Bill Fanslow, Xian Huang, Li Zhang, Hongyun Zhao

**Affiliations:** 1 Department of Medical Oncology, Sun Yat-sen University Cancer Center, State Key Laboratory of Oncology in South China, Collaborative Innovation Center for Cancer Medicine, Guangdong Key Laboratory of Nasopharyngeal Carcinoma Diagnosis and Therapy, No. 651 Dongfeng East Road, Guangzhou, 510060 China; 2 Department of Clinical Research, Sun Yat-sen University Cancer Center, State Key Laboratory of Oncology in South China, Collaborative Innovation Center for Cancer Medicine, Guangdong Key Laboratory of Nasopharyngeal Carcinoma Diagnosis and Therapy, No. 651 Dongfeng East Road, Guangzhou, 510060 China; 3 Department of Medical Oncology, Affiliated Hospital of Hebei University, Baoding, 071000 China; 4 Department of Thoracic Oncology, Jilin Cancer Hospital, Changchun, 130012 China; 5 The Cancer Hospital of the University of Chinese Academy of Sciences (Zhejiang Cancer Hospital), Institute of Basic Medicine and Cancer (IBMC), Chinese Academy of Sciences, Hangzhou, 310022 Zhejiang China; 6 Department of Oncology, First Affiliated Hospital of Gannan Medical University, Ganzhou, 341001 China; 7 Department of Medical Oncology, The Second Affiliated Hospital of Anhui Medical University, Hefei, 230093 China; 8 Department of Radiation Oncology, Guangxi Medical University Cancer Hospital, Cancer Institute of Guangxi, Nanning, 530021 Guangxi China; 9 Department of Medical Oncology of Respiratory, Guangxi Medical University Cancer Hospital, Cancer Institute of Guangxi, Nanning, 530021 Guangxi China; 10 Department of Head and Neck Radiation Oncology, Fujian Cancer Hospital, Fuzhou, 350000 China; 11 Department of Medical Oncology, Affiliated Cancer Hospital and Institute of Guangzhou Medical University, Guangzhou, 510095 China; 12 Rare Tumors Department, Shandong Cancer Hospital and Institute, Shandong First Medical University and Shandong Academy of Medical Sciences, Jinan, 250117 China; 13 Department of Oncology, Affiliated Hospital of Chengde Medical University, Chengde, 067000 China; 14 Department of Oncology, Second Xiangya Hospital, Central South University, Changsha, 410011 China; 15 Department of Medical Oncology, Affiliated Hospital of Inner Mongolia Medical University, Huhhot, 010050 Inner Mongolia China; 16 Tianjin Medical University Cancer Institute and Hospital, National Clinical Research Center for Cancer, Key Laboratory of Cancer Prevention and Therapy, Tianjin’s Clinical Research Center for Cancer, Tianjin, 300060 China; 17 Department of Oncology, Clinical Oncology Center, The People’s Hospital of Guangxi Zhuang Autonomous Region, Guangxi Academy of Medical Sciences, Nanning, 530021 China; 18 Department of Medical Oncology, Hainan General Hospital, Haikou, 570100 China; 19 Department of Medical Oncology, Yuebei People’s Hospital, Shaoguan, 512025 China; 20 Department of Otolaryngology & Head and Neck, The People’s Hospital of Guangxi Zhuang Autonomous Region, Guangxi Academy of Medical Sciences, Nanning, 530021 China; 21 Department of Medical Oncology, The First Affiliated Hospital of Nanchang University, Nanchang, 330000 China; 22 Department of Head and Neck/Thoracic Medical Oncology, The First People’s Hospital of Foshan, Foshan City, 528010 China; 23 Oncology Department, Liaocheng People’s Hospital, Liaocheng, 252004 China; 24 The Comprehensive Cancer Centre of Drum Tower Hospital, Medical School of Nanjing University and Clinical Cancer Institute of Nanjing University, Nanjing, 210008 China; 25 Department of Oncology, The Affiliated Cancer Hospital of Guizhou Medical University, Guiyang, 550001 China; 26 Department of Oncology, The First Affiliated Hospital of Kunming Medical University, Kunming, 650032 China; 27 Department of Oncology, The Affiliated Hospital of Qingdao University, Qingdao, 266003 China; 28 Clinical Research Center, Qilu Pharmaceutical Co., Ltd., Jinan, 250000 China; 29 Department of Clinical Pharmacology, Qilu Pharmaceutical Co., Ltd., Jinan, 250000 China; 30 Sound Biologics, 21720 23rd Drive SE, Suite200, Bothell, WA 98021 USA; 31 Department of Non-Clinical, Qilu Pharmaceutical Co., Ltd., Jinan, 250001 China

**Keywords:** Bifunctional PD-1, CTLA4 antibody, MabPair, Phase I trial, Nasopharyngeal carcinoma, Cervical cancer

## Abstract

**Background:**

QL1706 (PSB205) is a single bifunctional MabPair (a novel technical platform) product consisting of two engineered monoclonal antibodies (anti-PD-1 IgG4 and anti-CTLA-4 IgG1), with a shorter elimination half-life (t_1/2_) for CTLA-4. We report results from a phase I/Ib study of QL1706 in patients with advanced solid tumors who failed standard therapies.

**Methods:**

In the phase I study, QL1706 was administered intravenously once every 3 weeks at one of five doses ranging from 0.3 to 10 mg/kg, and the maximum tolerated dose, recommended phase 2 dose (RP2D), safety, pharmacokinetics (PK), and pharmacodynamics (PD) of QL1706 were investigated. In the phase Ib study, QL1706 was administered at the RP2D intravenously every 3 weeks, and the preliminary efficacies in non-small cell lung cancer (NSCLC), nasopharyngeal carcinoma (NPC), cervical cancer (CC), and other solid tumors were evaluated.

**Results:**

Between March 2020 and July 2021, 518 patients with advanced solid tumors were enrolled (phase I, *n* = 99; phase Ib, *n* = 419). For all patients, the three most common treatment-related adverse events (TRAEs) were rash (19.7%), hypothyroidism (13.5%), and pruritus (13.3%). The TRAEs and immune-related adverse events (irAEs) of grade ≥ 3 occurred in 16.0% and 8.1% of patients, respectively. In phase I, 2 of 6 patients in the 10mg/kg group experienced dose-limiting toxicities (DLTs) (grade 3 thrombocytopenia and grade 4 immune-mediated nephritis), so the maximum tolerated dose (MTD) was reached at 10 mg/kg. The RP2D was determined to be 5 mg/kg based on comprehensive analysis of tolerability, PK/PD, and efficacy. For all patients who received QL1706 at the RP2D, the objective response rate (ORR) and median duration of response were 16.9% (79/468) and 11.7 months (8.3—not reached [NR]), respectively; and the ORRs were 14.0% (17/121) in NSCLC, 24.5% (27/110) in NPC, 27.3% (15/55) in CC, 7.4% (2/27) in colorectal cancer, 23.1% (6/26) in small cell lung cancer. For immunotherapy-naive patients, QL1706 exhibited promising antitumor activities, especially in NSCLC, NPC, and CC, with ORRs of 24.2%, 38.7%, and 28.3%, respectively.

**Conclusions:**

QL1706 was well tolerated and demonstrated promising antitumor activity in solid tumors, especially in NSCLC, NPC, and CC patients. It is currently being evaluated in randomized phase II (NCT05576272, NCT05179317) and phase III (NCT05446883, NCT05487391) trials.

*Trial Registration* ClinicalTrials.gov Identifier: NCT04296994 and NCT05171790.

**Supplementary Information:**

The online version contains supplementary material available at 10.1186/s13045-023-01445-1.

## Background

Cytotoxic T-lymphocyte-associated antigen 4 (CTLA-4) and programmed cell death protein 1 (PD-1) are key immune checkpoint inhibitors of the T-cell immune response. The combination of anti-PD-1 and anti-CTLA-4 antibodies has been tested extensively in multiple tumor types in clinical trials [1–4]. The addition of an anti-CTLA-4 antibody to PD-1 blockade increases the objective response rate (ORR), which can often be translated into a longer duration of response (DoR) and survival [4, 5]. Thus, the combination of nivolumab and ipilimumab has been approved for the treatment of many advanced solid tumors such as melanoma, renal carcinoma, colorectal cancer (CRC), non-small-cell lung cancer (NSCLC), and hepatocellular carcinoma (HCC) [2, 6–8].

However, the combined inhibition of both PD-1 and CTLA-4 can lead to an increase of immune-related adverse events (irAEs) compared to anti-PD-1 monotherapy [9]. A previous study using different doses of nivolumab and ipilimumab in the combination therapy has demonstrated that the severity level of irAEs is more associated with the dose of ipilimumab than that of nivolumab [10], so the current strategy to manage the elevated toxicity is to reduce the dose and frequency of ipilimumab [11]. Nevertheless, the frequency of grade 3 or 4 adverse events (AEs) is still much higher with the combination therapy than with anti-PD-1 monotherapy [6]. Interestingly, in a recent study of quavonlimab (an anti-CTLA-4 IgG1 molecule) in combination with pembrolizumab (an anti-PD-1 antibody) in NSCLC patients, a low dose of CTLA-4 antibody (25 mg every 6 weeks plus 200 mg of anti-PD-1 every 3 weeks) demonstrated a better safety profile with an equal efficacy; therefore, this dose was selected as the recommended phase II dose (RP2D) for further studies [12, 13]. These findings suggest that there is additional room for improvement in terms of the safety and tolerability of the combination treatment. Another alteration of dual PD1 and CTLA4 blockade is the use of a bispecific antibody by binding two antigens or one antigen with different epitopes, which has demonstrated a promising efficacy [14]. However, the safety and efficacy of a bispecific antibody need to be further evaluated.

QL1706 was generated by using MabPair (patent No. US20190276542A1 in the USA, details in the Additional file 1), a new technological platform that enables the production of two antibodies close to their natural forms from a single host cell line and is manufactured as one product [15]. QL1706 contains a mixture of anti-PD-1 IgG4 and anti-CTLA-4 IgG1 that were produced together in a fixed ratio. Each antibody was individually optimized to achieve desirable target coverage and antibody effector functions. In particular, the anti-CTLA-4 antibody was engineered to have a shorter elimination half-life (t_1/2_) to reduce its exposure and lower the risk of irAEs (details in the Additional file 1). This unique profile of reduced anti-CTLA-4 exposure in the presence of a steady duration of anti-PD-1 exposure may improve tolerability and thus enable the patient to receive QL1706 for a longer period of time without discontinuation due to CTLA-4 antibody-mediated irAEs.

Based on this information, we conducted this phase I/Ib trial to investigate the safety, tolerability, pharmacokinetics (PK), pharmacodynamics (PD), and preliminary efficacy of QL1706 in patients with advanced solid tumors that have limited treatment options and poor survival after standard treatment.

## Methods

### Preclinical studies

The design and generation of QL1706 (PSB205) are presented in the Additional file 1: Methods. The MabPair cocktail was produced by multiple rounds of transient transfections in both Expi293 and ExpiCHO cells and purified with a protein A column. QL1706 was manufactured in a stable CHO cell line that was screened to produce anti-PD-1 IgG4 and anti-CTLA-4 IgG1 antibodies at an approximate ratio of 2:1. Mixed lymphocyte reaction and cytomegalovirus-specific CD8^+^ T-cell response assays were conducted. An in-vivo PK study of QL1706 was conducted in engineered nonobese diabetic/severe combined immunodeficiency gamma mice and protein-naive cynomolgus monkeys, respectively. The details are presented in the Additional file 1: Methods.

### Clinical study

#### Study design

This phase I/Ib, open-label, multicenter study consisted of a phase I study and a phase Ib study in patients with advanced solid tumors performed in 41 Grade A class 3 hospitals in China. Phase I was a dose escalation and expansion study. In the dose-escalation stage, accelerated titration (0.3 mg/kg) combined with the standard 3 + 3 design was adopted. The PK expansion stage planned to include 5–9 cases in selected cohorts. Five dose levels of QL1706 (0.3, 1.0, 3.0, 5.0, and 10.0 mg/kg) were administered every 3 weeks via intravenous infusion. The initial dose was determined by the minimum anticipated biologic effect level, the no-observed-adverse-effect level, and the preclinical data. Dose-limiting toxicities (DLTs) were evaluated in each cohort based on the QL1706-related AEs occurring within 21 days (1 cycle) after the administration of the first dose, which were defined as follows: grade 1, 3–5 nonhematologic AEs (G3 vomiting or nausea alleviated within 72 h and G3 fatigue were excluded); grade 2, 4–5 hematologic AEs; grade 3, thrombocytopenia with the symptom of bleeding; grade 4, febrile neutropenia; grade 5, any AEs leading to new treatment with steroids. Once the maximum tolerated dose (MTD) or maximum administered dose were reached, additional eligible patients were enrolled in the dose-expansion stage of phase I and treated with two dose levels of QL1706 chosen by the investigators to provide further evidence to establish the RP2D. The primary objectives of phase I were to determine the safety, tolerability (DLT and MTD), and RP2D of QL1706. Secondary objectives included immunogenicity, PK, and PD of QL1706.

In phase Ib, QL1706 was administered at the RP2D intravenously once every 3 weeks in patients with NSCLC, nasopharyngeal carcinoma (NPC), cervical cancer (CC), CRC, small cell lung cancer (SCLC), HCC, and other solid tumors. In principle, no fewer than 20 patients were included per tumor type, and the number of patients enrolled with each tumor type was adjusted in a timely manner based on the efficacy and safety results found during the research. The primary objective of phase Ib was to evaluate the preliminary efficacy of QL1706 in certain malignancies (NSCLC, NPC, CC, CRC, etc.) at the RP2D. Secondary objectives included safety and population-PK.

Each subject received QL1706 at only one dose level. A subject could be discontinued from the study for any of the following reasons: disease progression (unless the investigators believed that there was a continuous clinical benefit), study completion (up to 2 years), development of intolerable AEs, initiation of a new antitumor treatment, or informed consent withdrawal, whichever came first.

The study protocol and all amendments were approved by the Institutional Review Board of all participating institutions. All participants provided written informed consent. The study was done in accordance with Good Clinical Practice guidelines and the Declaration of Helsinki. This trial was registered at ClinicalTrials.gov (identifier: NCT04296994 and NCT05171790).

#### Patient population

Patients meeting the following key inclusion criteria were enrolled into phase I: (1) aged ≥ 18 years; (2) pathologically confirmed diagnosis of advanced solid tumor with failed on or with no standard antitumor therapy; (3) at least one measurable lesion according to RECIST v1.1; (4) with an Eastern Cooperative Oncology Group performance status of 0 or 1 and a life expectancy of greater than 3 months. The key exclusion criteria were as follows: (1) previous or active autoimmune disease, interstitial lung disease, or other diseases requiring long-term use of systemic corticosteroids (> 10 mg/day prednisone) or other immunosuppressive drugs; (2) grade 3 or 4 irAEs related to prior immunotherapy; (3) prior treatment with anti-CTLA-4 and anti-PD-(L)1 combination. Eligible patients received QL1706 as a single agent monotherapy.

In phase Ib (indication expansion), the types of tumors were specified for eligible patients, including pathologically confirmed metastatic or recurrent CC, ovarian cancer, fallopian tube cancer, endometrial carcinoma, NPC, gastric cancer, adenocarcinoma of esophago-gastric conjunction, esophageal carcinoma, and metastatic or recurrent solid tumors, including lung cancer (NSCLC, SCLC), HCC, cholangiocarcinoma, breast cancer, CRC, urothelial carcinoma, melanoma, and kidney cancer.

The major differences of phase Ib compared to phase I were that in phase Ib, the patients were required to have at least one measurable lesion according to the Response Evaluation Criteria In Solid Tumours (RECIST) v1.1, the absolute platelet count was set to > 75 × 10^9^/L, and the HCC patients should have Child–Pugh class A or B.

#### Safety assessments

The safety and tolerability were defined by the incidence of AEs and severe AEs. The grading of AEs was assessed by investigators according to the Common Adverse Event Evaluation Criteria, v5.0. The irAEs were mainly managed according to local medical practice. The investigator comprehensively evaluated the benefit/risk ratio of the subject and made a judgment on stopping/resuming dosing according to the management of the irAEs guideline [16].

#### Efficacy assessments

The efficacy was defined by the ORR, DoR, and disease control rate (DCR). The tumor response was assessed according to RECIST v1.1. Computed tomography scans or magnetic resonance images were performed at baseline, every two cycles (6 weeks) in the first four cycles, and every three cycles (9 weeks) thereafter.

#### PK and PD assessments

Plasma samples to characterize the systemic PK profiles of anti-CTLA-4 and anti-PD-1 components of QL1706 were collected in the dose-escalation stage of phase I. The data used for establishment of the population pharmacokinetic (PopPK) model were from patients not used for the systemic PK analysis. For PD assessment, the PD-1 receptor occupancy of QL1706 on human peripheral blood CD3^+^ T cells was calculated. The positive rates of Ki67 and inducible costimulator (ICOS) in human peripheral blood T cells were obtained. Detailed methods for PK, PD, and immunogenicity assessments can be found in the Additional file 1.

#### Evaluation of programmed death-ligand 1 (PD-L1) expression

PD-L1 expression was evaluated using immunohistochemistry (Ventana PD-L1, SP263). SP263 is a recombinant rabbit monoclonal antibody that binds to PD-L1 in paraffin-embedded tissue sections. Specific antibody localization can be performed by haptenated secondary antibodies in combination with multimeric anti-hapten-horseradish peroxidase (OptiVIEW DAB IHC Detection Kit). The specific antibody–enzyme complex is then visualized with a precipitating enzyme reaction product. The combined positive score (CPS) was used to evaluate the PD-L1 expression and was calculated as follows: the number of PD-L1-stained cells (tumor cells and related immune cells) divided by the total number of viable tumor cells, multiplied by 100. PD-L1 was considered to be expressed if the CPS was ≥ 1.

### Statistical analysis

The full analysis set included all patients enrolled in the study. The safety set included all patients who received at least one dose of QL1706. The PK analysis set consisted of patients in the safety set and had at least one evaluable PK sample. Efficacy analyses were those with at least one post-treatment image in the safety population. The data cutoff for this report was 2021-12-31. The ORR and DCR were calculated using the exact method. We calculated the DoR with the Kaplan–Meier method; median values and 95% confidence intervals (CIs) were calculated by the Brookmeyer–Crowley method. Comparisons between predose and postdose (cycle 1, 168 h) values for certain PD markers were made using the Wilcoxon signed-rank test. The analyses of demographics, safety, and tolerability were descriptive.

## Results

### Preclinical results

**Fig. 1:**
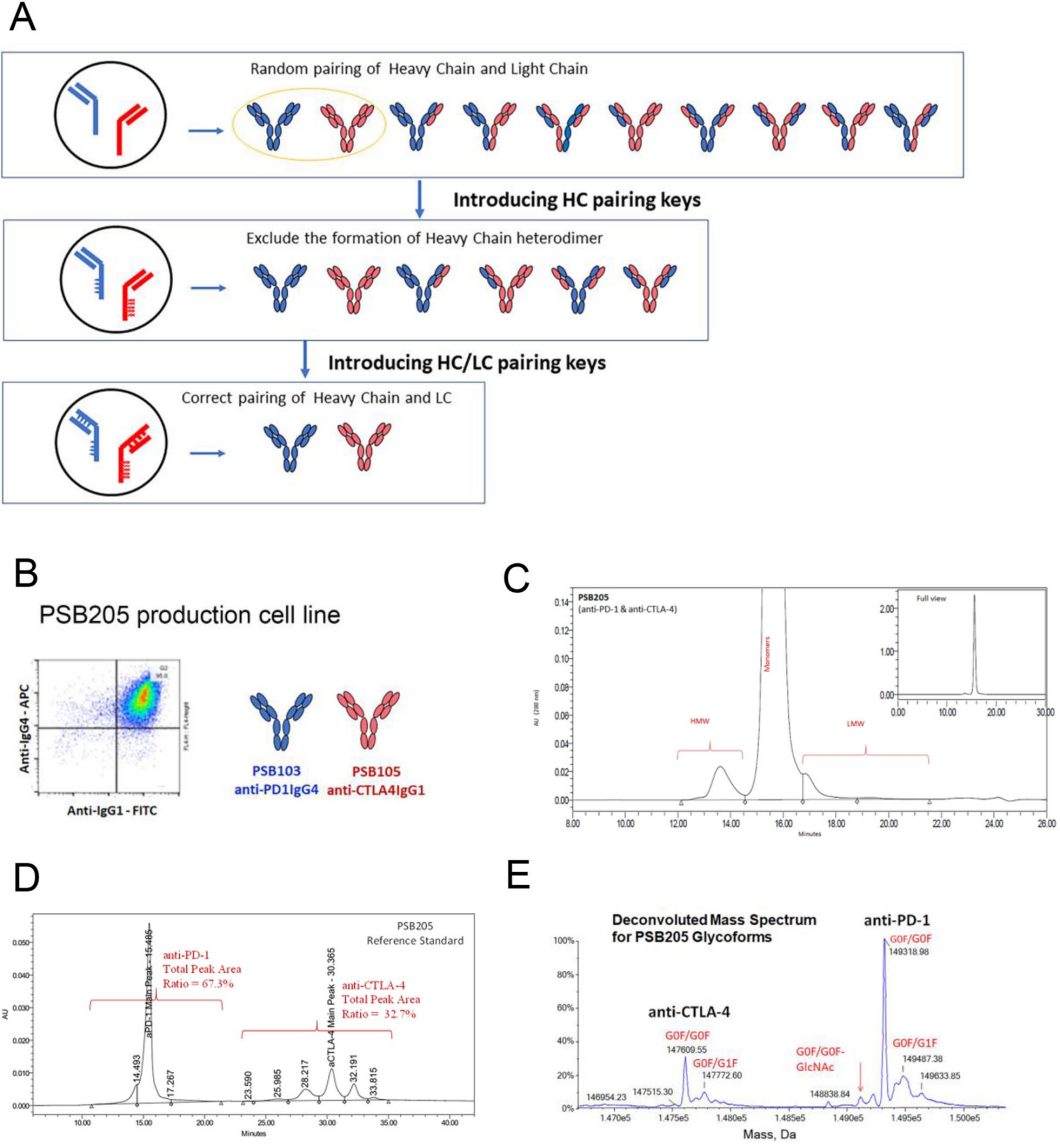
Generation and characterization of PSB205. **A** The principle of MabPair technology for producing two correctly assembled antibodies from a single mammalian cell line. Top panel, co-expression of two different antibodies in a single production cell line requires the simultaneous introduction of DNAs encoding two heavy chains (HCs) and two light chains (LCs) into the same cell. Under normal conditions, the two HCs can randomly dimerize to form two separate homodimers and one heterodimer species; the two LCs can also pair with either of the two HCs. The random combinations result in a total of 10 possible products generated, but only two of them are the desirable antibody products that contain the cognate HC/HC and LC/HC pairings (yellow-circled ones). Middle panel, using a charge-pair approach (referred as “HC pairing keys”) to correctly control the homodimeric pairing of the HCs of two different antibodies, four undesirable side products containing heterodimeric HCs are eliminated, so 10 combinations are reduced to 6. Bottom panel, applying a combined charge-pair and cysteine-pair approach (referred as “HC/LC pairing keys”) to control the cognate LC/HC pairings, only the two correctly paired and structurally stable products can pass the endogenous quality control system inside cells before they are secreted out. Other byproducts are fully eliminated due to their instability. **B** Fluorescence-assisted cell sorting plots showing the coexpression of PSB103 and PSB105 in the production cell line after intracellular staining of conjugated anti-hu IgG4- and anti-hu IgG1-specific antibodies, respectively. A panel of orthogonal analytical methods was used to characterize the PSB205 MabPair. The results confirmed the molecular integrity, and no mispaired antibodies were detected in the sequential characterization. **C** PSB205 size variants were analyzed by size-exclusion high-performance liquid chromatography (HPLC). The chromatogram shows the main peak for the monomers of the two mAbs overlaid, frontal minor peak(s) for high-molecular weight species, and post minor peak(s) for low-molecular weight species (not detected) in PSB205. As a result, the PSB205 purity as defined by the monomers (the main peak) was typically measured as 97–99% for different batches. **D** Baseline separation of the two mAbs in PSB205 was achieved by the hydrophobic interaction HPLC method. Thus, it served as a tool to determine the concentration ratio of the two mAbs, [anti-PD-1]:[anti-CTLA-4] (w/w). **E** The intact glycoform mass profile was obtained by liquid chromatography–mass spectrometry (LC–MS) analysis. As a result, the two main peaks at 149,320 Da and 147,610 Da in the deconvoluted mass spectra closely match the G0F/G0F glycoforms of anti-PD-1 and anti-CTLA-4, respectively, with their HC N-terminal Gln converted to pyroglutamic acid and the C-terminal Lys removed

In the preclinical study, PSB103 (anti-PD-1 IgG4) and PSB105 (anti-CTLA-4 IgG1) were produced together in the CHO cell line at a fixed ratio of 2:1 (Fig. 1A, B). PSB205 (QL1706) was manufactured by a novel antibody engineering technology platform and contains a mixture of these two recombinant antibodies as a MabPair molecule. Its purity and product quality were fully characterized by using a panel of analytical methods. No detectable mispairing species was found in the product (Fig. 1C–E). As displayed in Additional file 1: Fig. S1, PSB205 showed a satisfactory binding ability to block PD-1:PD-L1 and CTLA-4:B7-1/B7-2 interactions. Functional assessment of PSB205 indicated increased interferon gamma production by T cells and higher percentages and absolute numbers of cytomegalovirus-positive CD8-positive T cells were recovered from the PSB205-treated group (Additional file 1: Figs. S2, S3). A single mutation at arginine 255 was introduced in the Fc region of PSB105 to reduce the binding to FcRn (Additional file 1: Table S1), leading to a faster clearance and shortened t_1/2_ in vivo compared to ipilimumab (t_1/2_: 109 h vs. 397 h; clearance: 0.489 mL/h/kg vs. 0.0948 mL/h/kg; Additional file 1: Table S2). Preclinical experiments in humanized animal tumor models demonstrated antitumor activities of QL1706, with evidence of a functional dual blockade of both the PD-1 and CTLA-4 pathways (Additional file 1: Fig. S4). The detailed information is presented in the Additional file 1.

### Clinical results

#### Patient characteristics

**Fig. 2:**
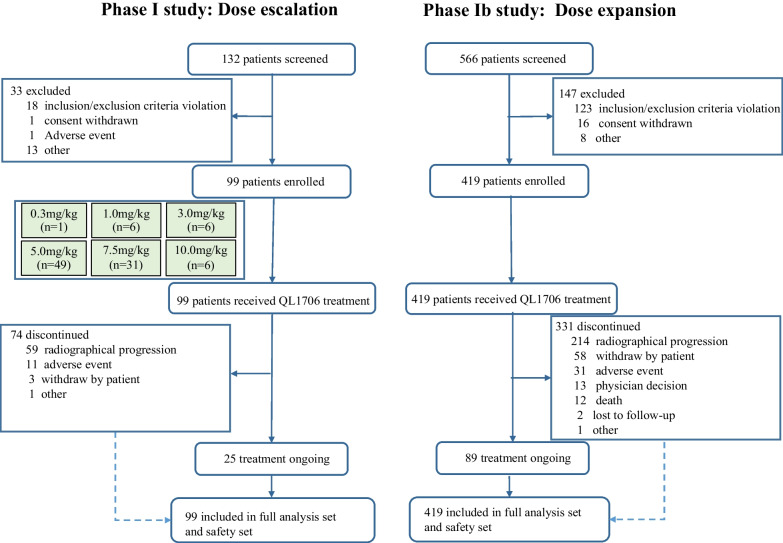
Flowchart of the study

A total of 518 patients were enrolled in this study, with 99 in phase I and 419 in phase Ib between March 2020 and July 2021 (Fig. 2). The baseline characteristics are shown in Table 1. The median age of the patients was 53 years old (range: 20–81 years old), and 180 (34.7%) patients had a history of immunotherapy. The tumor types were ranked as NSCLC (146, 28.2%), NPC (134, 25.9%), CC (55, 10.6%), CRC (27, 5.2%), SCLC (26, 5.0%), HCC (25, 4.8%), biliary tract carcinoma (21, 4.1%), breast cancer (19, 3.7%), kidney cancer (18, 3.5%), and other (39, 7.5%).

**Table 1:** Baseline characteristics of the included patients

Characteristic	Phase Ia	Phase Ib	Total
	(*n* = 99)	(*n* = 419)	(*n* = 518)
Age, median (years)	50	55	53
Range (years)	24–81	20–80	20–81
Sex, *n* (%)			
Male	74 (74.7)	244 (58.2)	318 (61.4)
Female	25 (25.3)	175 (41.8)	200 (38.6)
ECOG performance status, *n* (%)			
0	33 (33.3)	69 (16.5)	102 (19.7)
1	66 (66.7)	350 (83.5)	416 (80.3)
Tumor type, *n* (%)			
NSCLC	38 (38.4)	108 (25.8)	146 (28.2)
NPC	55 (55.6)	79 (18.9)	134 (25.9)
CC	0	55 (13.1)	55 (10.6)
CRC	0	27 (6.4)	27 (5.2)
SCLC	1 (1.0)	25 (6.0)	26 (5.0)
HCC	0	25 (6.0)	25 (4.8)
Biliary tract carcinoma	0	21 (5.0)	21 (4.1)
Breast cancer	0	19 (4.5)	19 (3.7)
Kidney cancer	0	18 (4.3)	18 (3.5)
Ovarian cancer	0	8 (2.0)	8 (1.5)
Other	5 (5.1)	34 (8.1)	39 (7.5)
Metastasis, *n* (%)			
No	0	8 (2.0)	8 (1.5)
Yes	99 (100.0)	411 (98.1)	510 (98.5)
History of immunotherapy, *n* (%)	46 (46.5)	134 (32.0)	180 (34.7)
Prior lines of therapy, *n* (%)			
0	2 (2.0)	22 (5.3)	24 (4.6)
1–2	59 (59.6)	261 (62.3)	320 (61.8)
3–4	30 (30.3)	93 (22.2)	123 (23.7)
≥ 5	8 (8.1)	40 (9.5)	48 (9.3)
Unknown	0	3 (0.7)	3 (0.6)

*ECOG* Eastern Cooperative Oncology Group, *NSCLC* Non-small-cell lung cancer, *NPC* Nasopharyngeal carcinoma, *CC* Cervical cancer, *SCLC* Small cell lung cancer, *CRC* Colorectal cancer, *HCC* Hepatocellular carcinoma

#### RP2D decision

Two patients receiving 10 mg/kg QL1706 experienced DLTs, and 7.5 mg/kg per 3 weeks was amended in the protocol as an additional escalation dose. As no DLTs occurred, 7.5 mg/kg per 3 weeks was the MTD. In phase I, the TRAEs (grade ≥ 3) of the 0.3-, 1-, 3-, 5-, 7.5-, and 10-mg/kg groups were 0%, 33.3%, 0%, 12.2%, 19.4%, and 50%, respectively. There were fewer severe TRAEs in the 5-mg/kg group compared to the 10-mg/kg group, and there was no significant dose-exposure correlation among doses of 1–7.5 mg/kg. The ORRs of the 0.3-, 1-, 3-, 5-, 7.5-, and 10-mg/kg groups were 0%, 33.3%, 16.7%, 22.9%, 10.7%, and 40% in phase I. In terms of PK, the 5-mg/kg group had a lower exposure level than the 7.5-mg/kg and 10-mg/kg groups. According to the PD analysis, Ki67 in CD8^+^ T cells in the 0.3-, 1-, 3-, 5-, and 10-mg/kg groups increased by 0.8%, 3.3%, 3.3%, 5.1%, and 10% compared with baseline, respectively. Ki67 in CD4^+^ T cells increased by − 1.5%, 3.5%, 5.1%, 8.1%, and 12%, respectively. The ICOS of CD4^+^ T cells in the 0.3–10-mg/kg groups increased by − 4.2%, 3.9%, 4.6%, 6.6%, and 10.3% compared with baseline, respectively. The activation and proliferation of T cells in the 5-mg/kg and 10-mg/kg groups were higher than those in the other dose groups. Based on the overall assessment of tolerability, safety, efficacy, PK, and PD in phase I, 5 mg/kg every 3 weeks was selected as the RP2D.

#### Safety

The median treatment duration was 2.1 (range: 0.03–19.8) months. Of all 518 patients in phase I and Ib, the treatment-emergent adverse events and treatment-related adverse events (TRAEs) are presented in Additional file 1: Table S3, Table 2. TRAEs of any grade occurred in 388 (74.9%) of the 518 patients, while TRAEs of grade ≥ 3 occurred in 16.0% of the total. Meanwhile, irAEs of any grade occurred in 239 (46.1%) patients, and irAEs of grade ≥ 3 occurred in 42 (8.1%) of the total (Table 2). The most common TRAEs were rash (19.7%), hypothyroidism (13.5%), pruritus (13.3%), increased aspartate aminotransferase (AST) level (11.0%), and fatigue (10.8%). Severe AEs occurred in 130 (25.1%) patients, and 64 (12.4%) were considered drug-related. Severe irAEs of any grade occurred in 47 (9.1%) of the patients, and severe irAEs of grade ≥ 3 occurred in 32 (6.2%) of the total. The most common severe irAEs were immune-mediated lung disease and immune-mediated myocarditis (Table 2). Dose interruption due to TRAEs occurred in 75 (14.5%) patients, and only 30 (5.8%) patients dropped out due to TRAEs (Table 2). Two patients in the 10-mg/kg group experienced DLTs, including one patient with grade-3 thrombocytopenia complicated with grade-1 gingival bleeding and another patient with grade-4 immune-mediated nephritis.

**Table 2:** Treatment-related adverse events in all treated patients

	Phase Ia (*n* = 99)		Phase Ib (*n* = 419)		Total (*n* = 518)	
	Any grade	Grade ≥ 3	Any grade	Grade ≥ 3	Any grade	Grade ≥ 3
TRAE	79 (79.8)	17 (17.2)	309 (73.7)	66 (15.8)	388 (74.9)	83 (16.0)
Severe TRAE	13 (13.1)	12 (12.1)	51 (12.2)	32 (7.6)	64 (12.4)	44 (8.5)
irAEs	60 (60.6)	9 (9.1)	179 (42.7)	33 (7.9)	239 (46.1)	42 (8.1)
Severe irAEs	10 (10.1)	10 (10.1)	37 (8.8)	22 (5.3)	47 (9.1)	32 (6.2)
TRAE leading to dose interruption	7 (7.1)	5 (5.1)	68 (16.2)	30 (7.2)	75 (14.5)	35 (6.8)
TRAE leading to drop out	10 (10.1)	10 (10.1)	20 (4.8)	16 (3.8)	30 (5.8)	26 (5.0)
TRAE leading to death	0	0	4 (1.0)	4 (1.0)	4 (0.8)	4 (0.8)
TRAEs occurring in ≥ 5% patients						
Rash	34 (34.3)	1 (1.0)	68 (16.2)	2 (0.5)	102 (19.7)	3 (0.6)
Hypothyroidism	22 (22.2)	0	48 (11.5)	0	70 (13.5)	0
Pruritus	33 (33.3)	0	36 (8.6)	0	69 (13.3)	0
AST increase	15 (15.2)	3 (3.0)	42 (10.0)	3 (0.7)	57 (11.0)	6 (1.2)
Fatigue	15 (15.2)	0	41 (9.8)	2 (0.5)	56 (10.8)	2 (0.4)
Hyperthyroidism	14 (14.1)	0	40 (9.5)	0	54 (10.4)	0
ALT increase	11 (11.1)	2 (2.0)	39 (9.3)	3 (0.7)	50 (9.7)	5 (1.0)
Pyrexia	6 (6.1)	0	40 (9.5)	0	46 (8.9)	0
Anemia	0	0	44 (10.5)	8 (1.9)	44 (8.5)	8 (1.5)
Appetite decrease	1 (1.0)	0	33 (7.9)	3 (0.7)	34 (6.6)	3 (0.6)
Lipase increase	5 (5.1)	1 (1.0)	27 (6.4)	3 (0.7)	32 (6.2)	4 (0.8)
Nausea	4 (4.0)	0	24 (5.7)	0	28 (5.4)	0
irAEs occurring in ≥ 5% patients						
Rash	34 (34.3)	1 (1.0)	49 (11.7)	2 (0.5)	83 (16.0)	3 (0.6)
Hypothyroidism	22 (22.2)	0	37 (8.8)	0	59 (11.4)	0
Pruritus	33 (33.3)	0	25 (6.0)	0	58 (11.1)	0
Hyperthyroidism	14 (14.1)	0	33 (7.9)	0	47 (9.1)	0
Severe irAEs occurring in > 1 patient						
Immune-mediated lung disease	2 (2.0)	2 (2.0)	8 (1.9)	5 (1.2)	10 (1.9)	7 (1.4)
Immune-mediated myocarditis	1 (1.0)	1 (1.0)	4 (1.0)	4 (1.0)	5 (1.0)	5 (1.0)
Infectious pneumonia	0	0	3 (0.7)	3 (0.7)	3 (0.6)	3 (0.6)
AST increase	2 (2.0)	2 (2.0)	1 (0.2)	0	3 (0.6)	2 (0.4)
Hepatic function abnormal	0	0	3 (0.7)	0	3 (0.6)	0
ALT increase	2 (2.0)	2 (2.0)	0	0	2 (0.4)	2 (0.4)
Immune-mediated hepatic disorder	0	0	2 (0.5)	2 (0.5)	2 (0.4)	2 (0.4)
Lipase increase	0	0	2 (0.5)	2 (0.5)	2 (0.4)	2 (0.4)
Immune-mediated myositis	0	0	2 (0.5)	1 (0.2)	2 (0.4)	1 (0.2)
Adrenal insufficiency	1 (1.0)	1 (1.0)	1 (0.2)	0	2 (0.4)	1 (0.2)
Platelet count decreased	0	0	2 (0.5)	1 (0.2)	2 (0.4)	1 (0.2)
Immune-mediated enterocolitis	0	0	2 (0.5)	0	2 (0.4)	0

*TRAE* Treatment-related adverse event, *irAE* Immune-related adverse event, *AST* Aspartate aminotransferase, *ALT* Alanine aminotransferase, *TSH* Thyroid-stimulating hormone, *GGT* Glutamyl transpeptidase

Of the 419 patients who received QL1706 at the RP2D in phase Ib, TRAEs occurred in 73.7% (308/419) of the patients, and 15.8% (66/419) were grade ≥ 3. The incidence rate of irAEs was 42.7% (179/419), and 30 patients (7.9%) experienced irAEs of grade ≥ 3. Severe TRAEs were observed in 51 patients (12.2%), and 32 of them (7.6%) were grade ≥ 3. The most common TRAEs were rash (16.2%), hypothyroidism (11.5%), anemia (10.5%), increased AST level (10.0%), and fatigue (9.8%). TRAEs leading to dose interruption occurred in 16.2% of the patients, and only 20 patients (4.8%) dropped out due to TRAEs. Four drug-related deaths occurred in phase Ib (infectious pneumonia, immune-mediated pneumonia, myocarditis, and hepatitis).

#### Efficacy analysis

The median follow-up time was 9.5 (95% CI 9.3–9.7) months. For all patients in phase I and Ib who received QL1706 at the RP2D (5 mg/kg every 3 weeks), the ORR and median DoR (mDoR) were 16.9% (79/468, 95% CI 13.6–20.6%) and 11.7 months (95% CI: 8.3–not reached [NR]) (Table 3). The Kaplan–Meier curve for the DoR of all patients receiving QL1706 at the RP2D is presented in Additional file 1: Fig. S6A. Among these patients, the ORR and mDoR were 14.0% (17/121, 95% CI 8.4–21.5%) and NR (95% CI 4.7–NR) in NSCLC, 24.5% (27/110, 95% CI 16.8–33.7%) and 11.7 months (95% CI 7.7–NR) in NPC, 27.3% (15/55, 95% CI 16.1–41.0%) and NR in CC, 7.4% (2/27, 95% CI 0.9–24.3%) and NR (95% CI 2.9-NR) in CRC, 23.1% (6/26, 95% CI 9.0–43.6%) and NR (95% CI 4.2–NR) in SCLC, 5.3% (1/19, 95% CI 0.1–26.1%) and NR in breast cancer, 27.8% (5/18, 95% CI 9.7–53.5%) and NR in kidney cancer, and 12.5% (1/8, 95% CI 0.3–52.7%) and NR in ovarian cancer (Table 3). The ORR was 0% (0/25) in HCC and 0% (0/21) in biliary tract carcinoma.

**Table 3:** Overall response of QL1706 in different tumor types in patients who received QL1706 at 5 mg/kg every 3 weeks (RP2D)

	All patient (*n* = 468)	NSCLC (*n* = 121)	NPC (*n* = 110)	CC (*n* = 55)	CRC (*n* = 27)	SCLC (*n* = 26)	HCC (*n* = 25)	Biliary tract carcinoma(*n* = 21)	Breast cancer (*n* = 19)	Kidney cancer (*n* = 18)	Ovarian cancer (*n* = 8)	Melanoma (*n* = 6)	Neuroendocrine tumor (*n* = 6)	Esophageal cancer (*n* = 5)	Gastric cancer (*n* = 3)	Others (*n* = 18)
CR	1 (0.2)	0	0	1 (1.8)	0	0	0	0	0	0	0	0	0	0	0	0
PR	78 (16.7)	17 (14.0)	27 (24.5)	14 (25.5)	2 (7.4)	6 (23.1)	0	0	1 (5.3)	5 (27.8)	1 (12.5)	2 (33.3)	2 (33.3)	0	0	1 (5.6)
SD	129 (27.6)	37 (30.6)	27 (24.5)	14 (25.5)	5 (18.5)	3 (11.5)	10 (40.0)	9 (42.9)	4 (21.1)	8 (44.4)	3 (37.5)	1 (16.7)	2 (33.3)	2 (40.0)	0	4 (22.2)
PD	213 (45.5)	52 (43.0)	52 (47.3)	18 (32.7)	18 (66.7)	11 (42.3)	15 (60.0)	12 (57.1)	12 (63.2)	4 (22.2)	3 (37.5)	1 (16.7)	1 (16.7)	2 (40.0)	3 (100)	9 (50.0)
NE	2 (0.4)	0	0	1 (1.8)	1 (3.7)	0	0	0	0	0	0	0	0	0	0	0
ND	45 (9.6)	15 (12.4)	4 (3.6)	7 (12.7)	1 (3.7)	6 (23.1)	0	0	2 (10.5)	1 (5.6)	1 (12.5)	2 (33.3)	1 (16.7)	1 (20.0)	0	4 (22.2)
Confirmed ORR, *n* (%, 95% CI)	79 (16.9, 13.6–20.6)	17 (14.0, 8.4–21.5)	27 (24.5, 16.8–33.7)	15 (27.3, 16.1–41.0)	2 (7.4, 0.9–24.3)	6 (23.1, 9.0–43.6)	0	0	1 (5.3, 0.1–26.1)	5 (27.8, 9.7–53.5)	1 (12.5, 0.3–52.7	2 (33.3, 4.3–77.7)	2 (33.3, 4.3–77.7)	0	0	1 (5.6, 0.1–27.3)
DCR, *n* (%, 95% CI)	208 (44.4, 39.9–49.1)	54 (44.6, 35.6–53.9)	54 (49.1, 39.4–58.8)	29 (52.7, 38.8–66.3)	7 (25.9, 11.1–46.3)	9 (34.6, 17.2–55.7)	10 (40.0, 21.1–61.3)	9 (42.9, 21.9–66.0)	5 (26.3, 9.1–51.2)	13 (72.2, 46.5–90.3)	4 (50, 15.7–84.3)	3 (50.0, 11.8–88.2)	4 (66.7, 22.3–95.7)	2 (40.0, 5.3–85.3)	0	5 (27.8, 9.7–53.4)
Number of DoR events (%)	20 (25.3)	5 (29.4)	9 (33.3)	2 (13.3)	1 (50.0)	1 (16.7)	/	/	1 (100)	0	/	1 (50.0)	0	/	/	0
DoR, m (95% CI)	11.7 (8.3, –)	− (4.7, –)	11.7 (7.7, –)	− (–, –)	− (2.9, –)	− (4.2, –)	/	/	/	− (–, –)	/	− (5.4, –)	− (–, –)	/	/	/

*CRC* Colorectal cancer, *SCLC* Small cell lung cancer, *HCC* Hepatocellular carcinoma, *CR* Complete response, *PR* Partial response, *SD* Stable disease, *PD* Progressive disease, *NE* Not evaluated, *ND* Not determined, *ORR* Objective response rate, *DCR* Disease control rate, *CI* Confidence interval

**Fig. 3:**
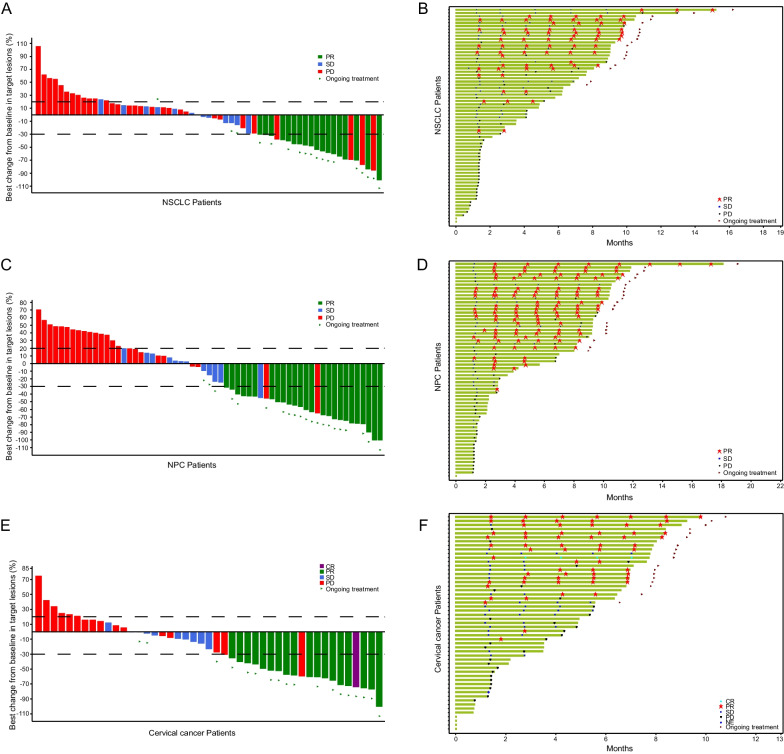
Tumor response. The waterfall plot (**A**, **C**, **E**) of the best overall responses with respect to the tumor size and the swimmer plot (**B**, **D**, **F**) of time to tumor response in non-small-cell lung cancer (NSCLC) patients (**A**, **B**), nasopharyngeal carcinoma (NPC) patients (**C**, **D**), and cervical cancer (CC) patients (**E**, **F**)

For immunotherapy-naive patients treated with QL1706 at the RP2D, the ORR and mDoR were 24.2% (16/66, 95% CI 14.5–36.4%) and NR (95% CI 3.5–NR) in NSCLC, 38.7% (24/62, 95% CI 26.6–51.9%) and NR (95% CI 7.7–NR) in NPC, and 28.3% (15/53, 95% CI 16.8–42.3%) and NR in CC, respectively. The Kaplan–Meier curves for the DoR of immunotherapy-naive NSCLC, NPC, and CC patients are presented in Additional file 1: Fig. S6B-D. For immunotherapy-treated patients, the ORR and mDoR were 1.8% (1/55, 95% CI 0.04–9.7%) and NR in NSCLC, and 6.3% (3/48, 95% CI 1.3–17.2%) and 11.7 months (95% CI 5.9–11.7) in NPC, respectively (Table 4). The best overall responses of the target lesions from baseline and the duration of treatment for the immunotherapy-naive NSCLC, NPC, and CC patients are shown in Fig. 3.

**Table 4:** Overall response based on prior immunotherapy history in patients who received QL1706 at 5 mg/kg every 3 weeks (RP2D)

	All patients (*n* = 468)		Primary tumor type					
			NSCLC (*n* = 121)		NPC (*n* = 110)		CC (*n* = 55)	
	Immunotherapy-naive patients (*n* = 312)	Immunotherapy-treated patients (*n* = 156)	Immunotherapy-naive patients (*n* = 66)	Immunotherapy-treated patients (*n* = 55)	Immunotherapy-naive patients (*n* = 62)	Immunotherapy-treated patients (*n* = 48)	Immunotherapy-naive patients (*n* = 53)	Immunotherapy-treated patients (*n* = 2)
CR, *n* (%)	1 (0.3)	0	0	0	0	0	1 (1.9)	0
PR, *n* (%)	71 (22.8)	7 (4.5)	16 (24.2)	1 (1.8)	24 (38.7)	3 (6.3)	14 (26.4)	0
SD, *n* (%)	81 (26.0)	48 (30.8)	15 (20.7)	22 (40.0)	13 (21.0)	14 (29.2)	14 (26.4)	0
PD, *n* (%)	133 (42.6)	80 (51.3)	32 (48.3)	20 (36.4)	24 (38.7)	28 (58.3)	16 (30.2)	2 (100)
NE, *n* (%)	2 (0.6)	0	0	0	0	0	1 (1.9)	0
ND, *n* (%)	24 (7.7)	21 (13.5)	3 (5.2)	12 (21.8)	1 (1.6)	3 (6.3)	7 (13.2)	0
Confirmed ORR, *n* (%, 95% CI)	72 (23.1, 18.5–28.1)	7 (4.5, 1.8–9.0)	16 (24.2, 14.5–36.4)	1 (1.8, 0.04–9.7)	24 (38.7, 26.6–51.9)	3 (6.3, 1.3–17.2)	15 (28.3, 16.8–42.3)	0
DCR, *n* (%, 95% CI)	153 (49.0, 43.3–54.7)	55 (35.3, 27.8–43.3)	31 (47.0, 34.6–60.0)	23 (41.8, 28.7–55.9)	37 (59.7, 46.5–71.9)	17 (35.4, 22.2–50.5)	29 (54.7, 40.4–68.4)	0
Number of DoR events (%)	16 (22.2)	4 (57.1)	4 (25.0)	1 (100)	7 (29.2)	2 (66.7)	2 (13.3)	/
DoR, m (95% CI)	− (8.1, –)	11.7 (4.2, 11.7)	− (3.5, –)	/	− (7.7, –)	11.7 (5.9, 11.7)	− (–, –)	/

*NSCLC* Non-small-cell lung cancer, *NPC* Nasopharyngeal carcinoma, *CC* Cervical cancer, *CR* Complete response, *PR* Partial response, *SD* Stable disease, *PD* Progressive disease, *NE* Not evaluated, *ND* Not determined, *ORR* Objective response rate, *DCR* Disease control rate, *CI* Confidence interval, *DoR* Duration of response

A total of 112 patients receiving the RP2D had PD-L1 data. For the 76 patients with CPS ≥ 1, the ORR was 27.6% (21/76) compared to 16.7% (6/36) for 37 patients with CPS < 1 (Additional file 1: Table S4). For NSCLC, the ORR was 29.4% (5/17) in 17 patients with CPS ≥ 1 and 12.5% (1/8) in 8 patients with CPS < 1. For CC patients with CPS ≥ 1, the ORR was 36.4% (8/22) compared to 25.0% (2/8) in those with CPS < 1 (Additional file 1: Table S4).

#### PK and PD analyses

**Fig. 4:**
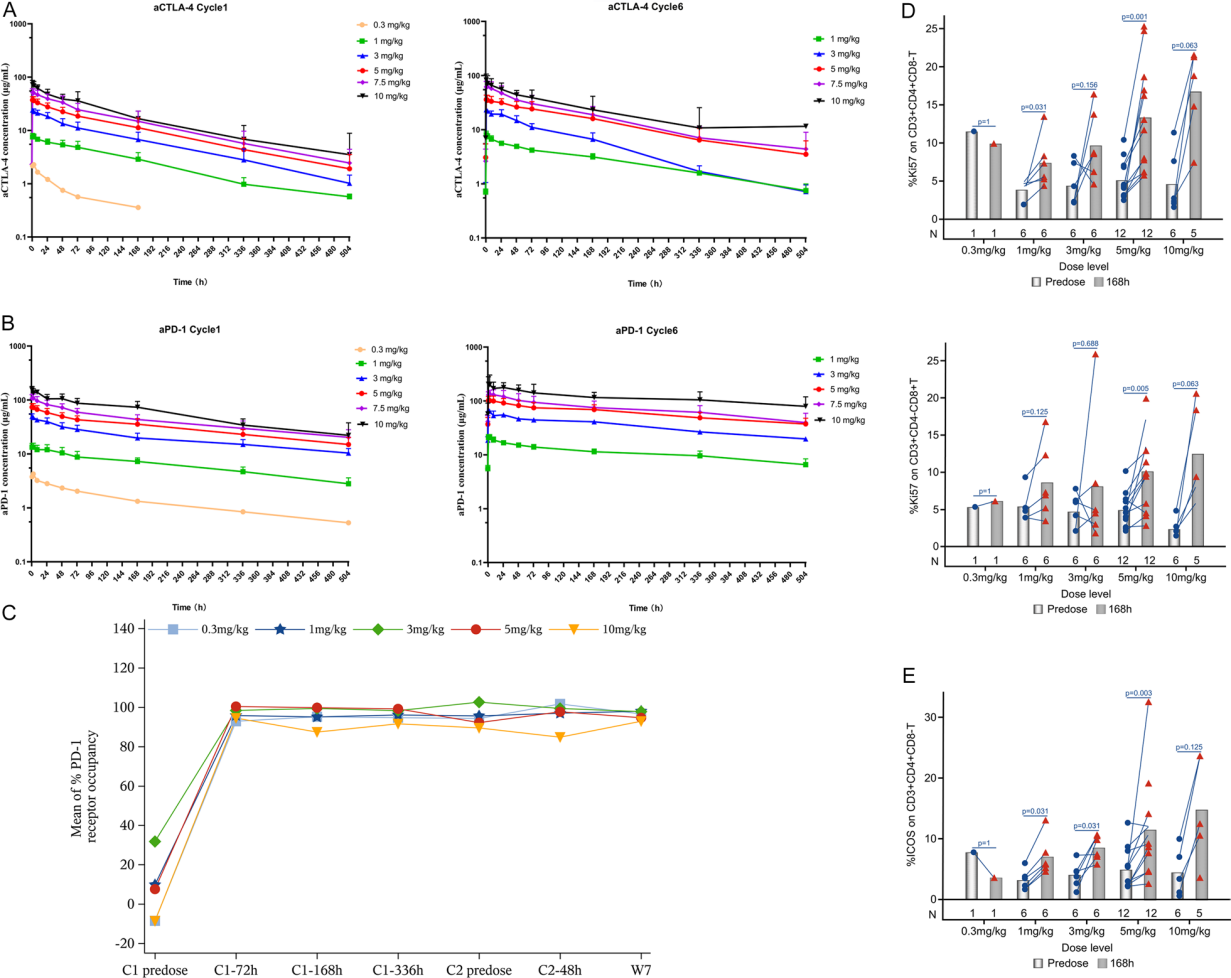
Mean (± standard deviation) plasma concentrations of anti-CTLA-4 (**A**) and anti-PD-1 (**B**) as a function of time following dosing in cycle 1 and at steady state (cycle 6) shown on a log10 scale in μg/mL across dose levels from 0.3 mg/kg to 10.0 mg/kg Q3W. **C** Mean % PD-1 receptor occupancy. Expression changes of Ki67 (**D**) and ICOS (**E**) on T cells in each dose group (Note, the values out of the visit window range or deviated from the protocol were not included in the summary analysis. When more than half (> 50%) of the values at a single time point are below the quantization limit (BQL), the mean values are reported as 0. For those BQL values, they are omitted on the semi-log scale plot. When there are only two samples at a single time point, the error bars are not presented

The PK profile of the anti-PD-1 and anti-CTLA-4 components of QL1706 were characterized separately by using two different anti-idiotypic antibodies that are specific to each component. The exposure of both anti-CTLA-4 and anti-PD-1 increased as the dose increased following single and multiple dosing (Fig. 4A, B). Anti-CTLA-4 and anti-PD-1 showed basically linear PK characteristics, which are summarized in Additional file 1: Table S5. For the anti-CTLA-4 component, the mean t_1/2_ values were 112–121 h (4.7–5 days) following a single dose and 112–190 h (4.7–7.9 days) following multiple doses. For the anti-PD-1 component, the mean t_1/2_ value was 175–293 h (7.3–12.2 days) following a single dose, and it increased to 341–477 h (14.2–19.9 days) following multiple doses. A two-compartment PopPK model with time-dependent elimination was established to best describe the anti-PD-1 component, while a PopPK model with linear elimination was established for the anti-CTLA-4 component (Additional file 1: Tables S6, S7). The effect of all the significant covariates was less than 25% on the steady-state exposure parameters of anti-PD-1 and was less than 37% on those of anti-CTLA-4 compared with typical individuals dosed at 5 mg/kg Q3W (Additional file 1: Fig. S5), suggesting that there were no significant differences clinically. More details are included in the Additional file 1.

For PD analysis, the level of PD-1 target coverage was assessed by a receptor occupancy assay on circulating CD3^+^ T cells. A sustained high PD-1 receptor occupancy rate (> 90%) was observed in all dosing groups throughout treatment (Fig. 4C). No dose-dependent difference in receptor occupancy was observed. QL1706 administration was associated with the enhanced proliferation of CD8^+^ cells. As depicted in Fig. 4D, Ki67 in CD8^+^ and CD4^+^ T cell elevation to baseline increased along with the QL1706 dose (0.3–10 mg/kg), which was also observed in the ICOS of CD4^+^ T cells (Fig. 4E). There was a dose-dependent upregulation of T cell activation and proliferation activity.

## Discussion

To the best of our knowledge, this is the first phase I clinical trial of a bifunctional MabPair product providing dual inhibition of PD-1 and CTLA-4 (QL1706), and it is the largest phase I trial of a dual immune checkpoints inhibitor. QL1706 was well tolerated, with only 83 out of 518 (16%) participants experiencing TRAEs of grade ≥ 3. In addition, it exhibited good antitumor activity in multiple cancer types including NSCLC, NPC, CC, and SCLC, especially in heavily pretreated immune-naive NPC and cervical cancer patients, with ORRs of 38.7% and 28.3% and the DoR not researched in the median follow-up time of 10.9 (95% CI 10.7–11.4) and 7.5 (95% CI 7.2–8.2) months, respectively.

In contrast to a bispecific antibody (i.e., KN046 and cadonilimab) that covers the two targets of PD-1 and CTLA-4 equally [17–19], QL1706 enables its two antibody components to provide a distinct target-specific level of PK coverage and antibody effector function. This was achieved by adjusting the ratio in which the two antibodies are produced together in the CHO cell line and the PK profile of each antibody. The Fc-mediated effector mechanism of anti-CTLA-4 IgG1 can improve the priming of the T-cell response and increase the diversity of T-cell clones, which may help to bring new T cells to the tumor microenvironment [20]. However, prolonged T-cell expansion can lead to immune-related toxicity [21]. The anti-CTLA-4 IgG1 of QL1706 was engineered to reduce binding to FcRn, leading to a faster clearance in the circulation and a shorter t_1/2_ (about 5 days in humans), which is significantly shorter than that of ipilimumab (> 12.5 days) [22]. These unique features may allow more flexibility in drug exposure and better tolerance.

As shown in this study, QL1706 is generally well tolerated, and TRAEs of grade ≥ 3 occurred in 16.0% of the patients. Previous studies have shown that the pooled incidence of TRAEs of grade ≥ 3 with the combination treatment of nivolumab and ipilimumab in advanced malignancies was 39.9% [23], 32.8% in NSCLC [6], 22% in microsatellite instability-high/mismatch repair-deficient CRC [24], 29–53% in HCC [8], 30.3% in malignant pleural mesothelioma [25], 46% in renal cell carcinoma [26], and 59% in melanoma [27]. In the first clinical trial of nivolumab and ipilimumab combination therapy, 29.4% patients discontinued due to the irAEs in the combination arm compared to 5.1% for nivolumab and 13.2% for ipilimumab [28]. Later, researchers tried to reduce the irAEs by adjusting the dosage and dose interval of ipilimumab, from 3 to 1 mg/kg Q3W, and then 1 mg/kg Q6W, while maintaining the efficacy of the combination therapy [29]. The overall safety profile of QL1706 compares favorably to the published data of anti-PD-1 and anti-CTLA-4 antibodies used in combination. QL1706 may reduce the discontinuation rate and can potentially prolong the duration of treatment. In this study, four drug-related deaths (0.7%, 4/518) occurred. A previous pooled analysis safety profile of nivolumab and ipilimumab combination therapy reported that 2.0% (31/2536) [23] of patients experienced drug-related deaths. Although TRAEs of grade ≥ 3 were numerically low, the data should be interpreted with caution due to the limitations of cross-trial comparison. Severe irAEs should be noticed and managed according to the guidelines for irAEs during treatment.

The efficacy of QL1706 in patients with diverse tumor types was investigated in 468 patients, and 16.9% (79/ 468) had objective responses with durability. ORRs of 12.5–33% were observed in NSCLC, NPC, CC, SCLC, kidney cancer, melanoma, and neuroendocrine tumors. Based on the preliminary results, the efficacy of QL1706 in these tumor types deserved further evaluation. QL1706 demonstrated limited efficacy in CRC, HCC, biliary tract carcinoma, breast cancer, esophageal cancer, and gastric cancer. Though no conclusion can be drawn with such a small sample size, the mechanisms of poor efficacy need to be explored to guide further drug development.

In immunotherapy-naive NPC patients, the ORR of QL1706 was 38.7%, and the mDoR was NR (> 7.7 months). The ORR was relatively greater than that of other PD-1 antibodies, like nivolumab (ORR: 20.5%) [30], pembrolizumab (ORR: 25.9%) [31], camrelizumab (ORR: 28.2%), and toripalimab (ORR: 23%) for prior-treated recurrent or metastatic NPC [32, 33]. The promising high response rate observed for QL1706 in NPC is likely attributable to the contribution of the anti-CTLA-4 component, since NPC patients have been shown to have elevated infiltration of regulatory T cells in the tumor [34] and might be more sensitive to treatment with anti-CTLA-4 antibodies. In addition, for immunotherapy-treated NPC patients, QL1706 also demonstrated antitumor activities, with the mDoR as long as 11.7 months, which was very significant for these PD-1-refractory patients. The encouraging data observed in the present study warrant further clinical studies in NPC patients, and a phase III trial of QL1706 combined with chemotherapy (NCT05576272) is ongoing.

At present, there are limited treatment options for patients with advanced CC and SCLC, especially for pretreated ones. A phase II study of pembrolizumab for patients with pretreated advanced cervical cancer achieved an ORR of 12.2% [35]. Meanwhile, the anti-PD-1 antibody balstilimab alone resulted in an ORR of 15% [36]. The ORR for QL1706 in advanced pretreated cervical cancer patients was 27.3% (15/55), and the mDoR was NR, with a median follow-up time of 7.5 (95% CI 7.2–8.2) months, which is comparable to dual PD-1 and CTLA-4 checkpoint blockade using the combination of balstilimab and zalifrelimab, which led to an ORR of 25.6% [37]. These findings indicate that the combination of PD-1 and CTLA-4 checkpoint blockade may be a promising therapy for advanced cervical cancer. Phase II (NCT05179317) and phase III (NCT05446883) clinical trials of cervical cancer are ongoing. The ORRs of pembrolizumab and nivolumab in patients with recurrent or metastatic SCLC after two or more lines of previous therapy were 19.3% and 11.9%, respectively [38, 39]. QL1706 achieved an ORR of 23.1% (6/26) for these patients in our study, which is very promising and deserves further investigation.

In the present study, the promising efficacy of QL1706 may be independent of the expression of PD-1, since patients with CPS < 1 also showed a relatively high response. However, due to the small sample size, further clinical trials with a larger sample size are needed to confirm this hypothesis.

This study has some limitations that must be addressed, including the absence of randomization in phase Ib and the lack of quality-of-life outcome evaluations in our analyses. Moreover, only Chinese patients were enrolled. The efficacy of QL1706 on other ethnic populations warrants further trials.

In conclusion, QL1706, the first-in-class bifunctional MabPair product with dual blockade of PD-1 and CTLA-4 showed a good safety profile and encouraging antitumor activity in this large-sample phase I study of advanced solid tumors. It is a potential new option for cancer dual immunotherapy and a backbone agent for combined therapy, which needs to be further developed.

## Supplementary Information

**Additional file 1.** Supplementary data of first-in-human phase I/Ib study of QL1706 (PSB205) in patients with advanced solid tumors.

## Data Availability

The datasets generated and analyzed during the current study are available at the following research data deposit public platform: www.researchdatadeposit.com (ID: RDDA2022217090). All requests should be submitted to the corresponding authors and are available on reasonable request.

## Abbreviations

CTLA-4: Cytotoxic T-lymphocyte-associated antigen 4
PD-1: Programmed cell death protein 1
ORR: Objective response rate
DoR: Duration of response
mDoR: Median duration of response
CRC: Colorectal cancer
NSCLC: Non-small-cell lung cancer
HCC: Hepatocellular carcinoma
SCLC: Small cell lung cancer
ICOS: Inducible costimulator
irAE: Immune-related adverse event
AE: Adverse event
RP2D: Recommended phase II dose
DLT: Dose-limiting toxicity
NPC: Nasopharyngeal carcinoma
CC: Cervical cancer
VH: Variable heavy
VL: Variable light
MTD: Maximum tolerated dose
DCR: Disease control rate
PopPK: Population pharmacokinetics
Cmax: Maximum concentration
Tmax: Time to maximum concentration
AUC: Area under the time–concentration curve
CL: Clearance
PD-L1: Programmed death-ligand 1
CI: Confidence interval
DC: Dendritic cell
TRAE: Treatment-related adverse event
NR: Not reached
PK: Pharmacokinetics
PD: Pharmacodynamics
CPS: Combined positive score
AST: Aspartate aminotransferase
