# Supplementary material for: First-in-human phase I/Ib study of QL1706 (PSB205), a bifunctional PD1/CTLA4 dual blocker, in patients with advanced solid tumors
Source: J Hematol Oncol. 2023 May 8;16:50. doi: 10.1186/s13045-023-01445-1 (PMC10169367; doi:10.1186/s13045-023-01445-1)
Supplement: Supplementary file 1 — Additional file 1. Supplementary data of first-in-human phase I/Ib study of QL1706 (PSB205) in patients with advanced solid tumors. [file 13045_2023_1445_MOESM1_ESM.docx]

# Supplementary Materials and Methods

***Preclinical studies***

## Design and generation of PSB205 (QL1706)

The generation and engineering of anti-human PD-1 antibody clone #1 and the anti-human CTLA-4 antibody 11F4 have been described previously (US2019/0248899 and US2019/0276542). The variable heavy (VH) and variable light (VL) chains of anti-PD-1 were inserted into a human gamma-4 constant HC and a constant kappa LC, respectively. The substitution S228P at the hinge region was introduced to prevent the Fab arm exchange of IgG4. The VH and VL of anti-CTLA-4 were inserted into a human gamma-1 constant HC and a constant kappa LC, respectively. Several substitutions were introduced in anti-CTLA-4 IgG1 antibody to precisely control the cognate HC/HC and HC/LC chain pairings when co-expressed with the anti-PD-1 IgG4 antibody in the same cells. In addition, one substitution (R255K) in the C_H_2 region was introduced to alter the binding of FcRn.

The MabPair cocktail was produced by multiple rounds of transient transfections in both Expi293 and ExpiCHO cells and purified with a protein A column. Mass spectral analysis confirmed that all HC/HC and HC/LC chains were correctly assembled without any mispairings.

## Production of PSB205 (QL1706) in a stable CHO cell line

The DNAs encoding both the HC and LC of anti-PD-1 IgG4 antibody were subcloned in the pCHO1.0 vector (Thermo Fisher) and used for the transfection and selection of the CHO-S^TM^ cell line. One stable cell line producing a high level of anti-PD-1 IgG4 antibody, clone G19G4-4B4, was selected as the host cell for introducing the LC and HC of engineered anti-CTLA-4 IgG1 antibody. Stable clones with a high expression titer of both antibodies were further screened to identify a single clone of a CHO cell that can produce anti-PD-1 IgG4 and anti-CTLA-4 IgG1 antibodies at an approximate ratio of 2:1.

## Mixed lymphocyte reaction

Monocytes were cultured in the presence of IL-4 and GM-CSF for 6 days. Immature DCs were harvested and stored in liquid. A total of 2×10^6^ cells were thawed on the day of the experiment. T cells were purified by using a Miltenyi Pan T cell isolation kit. A total of 2.2×10^6^ T cells were used for the experiment. The experiment was performed in a 96-well U-bottom plate. DCs and T cells were mixed at ratios of 1:10 and 1:3 in 200 μL of RPMI + 10% fetal calf serum + 50 μM 2-mercaptoethanol. Serial dilutions (ten-fold) of antibodies were added to the culture (range: 0.0068–68 nM): PSB205 (10 μg/mL; 68 nM), PSB103 (anti-PD-1 start: 7 μg/mL; 47 nM), PSB105 (anti-CLTA4 start: 3 μg/mL; 20 nM), and hIgG1 (10 μg/mL; 68 nM). Each dose was tested in triplicate. At day 6 after stimulation, the supernatant was collected, and the IFN-γ levels were evaluated by ELISA (R&D Systems).

## CMV-specific CD8^+^ T cell response

The stimulation was performed in a 48-well plate with 200 μL of complete medium. A total of 3.8×10^6^ CD8^+^ T cells, purchased from BenTech (Seattle, WA, USA), in each well was stimulated with CMV lysate (3 μg/mL) in the presence of various antibodies in duplicate: IgG1 (5 μg/mL), PSB103 (5 μg/mL), PSB105 (2.5 μg/mL), and PSB205 (7.5 μg/mL). At day 7 after stimulation, the cells were collected and stained with 2 μL of Dextramer HLA-A*0201/NLVPMVATA/PE for 15 min at room temperature followed by 2 μL of anti-CD8 for 30 min at room temperature. Day 7 was found to be the optimal time to allow the T cell receptor to recover after initial downregulation. After washing three times with phosphate-buffered saline (PBS), the cell pellet was resuspended in 400 mL of PBS/bovine serum albumin. All cellular contents in each tube were analyzed and enumerated using an LSR II flow cytometer.

## In-vivo assay

The engineered NCG mice were inoculated subcutaneously in the right flank region with HCC827 tumor cells (5×10^6^) in 0.1 mL of PBS for tumor development. A total of 1×10^7^ PBMCs (100 μL) was implanted intravenously into each mouse when the mean tumor size reached 60–80 mm^3^ (~5 days post tumor inoculation). The treatments started at 1 h after PBMC implantation. The mice were randomized into four groups (*n*=5 mice/group), each of which received an intraperitoneal dose of human IgG1 control (7.5 mg/kg), PSB103 (5 mg/kg), PSB105 (2.5 mg/kg), or PSB205 (7.5 mg/kg), twice per week (BIW) for 3 weeks. The date of tumor cell inoculation was denoted as day 0. Before grouping and treatment, all animals were weighed, and the tumor volumes were measured using a caliper. The tumor volumes and body weight were measured postadministration to monitor the antitumor efficacy and gross tolerability. The tumor growth inhibition and tumor volume were used as the pharmacodynamics markers in this study.

## Pharmacokinetic study in animals

Two single-dose studies were conducted in protein-naive cynomolgus monkeys to independently evaluate the PK profile of each PSB205 component in the absence of interference with the other component. PSB103 and human IgG4 (1/sex/group, 5 mg/kg) were examined in the first study, while PSB105, human IgG1, and ipilimumab (1/sex/group, 3 mg/kg) were investigated in the second study. In one study, four protein-naive cynomolgus monkeys (1/sex/group) received a single dose of 5 mg/kg PSB103 or human IgG4 via an intravenous bolus injection. Blood samples for pharmacokinetic measurements were collected from individual animals at predose as well as 0.083 h, 0.5 h, 2 h, 8 h, 16 h, 24 h, 72 h, 144 h, 240 h, 336 h, 504 h, and 672 h (28 days) postdose. In the second single-dose PK study, six protein-naive cynomolgus monkeys (1/sex/group) received a single dose of 3 mg/kg PSB105 (10511), 10511P, or ipilimumab (10D1) via i.v. bolus injection. 10511P is the parental control antibody that contains the wild-type sequence in the FcRn binding region, while PSB105 (10511) possesses the introduced R225K mutation to reduce FcRn binding. PK blood samples were collected from individual animals at predose, and 0.083 h (5 min), 0.5 h, 2 h, 8 h, 16 h, 24 h, 72 h, 144 h, 240 h, 336 h, 504 h, and 672 h (28 days) postdose. The serum concentration of the tested substance over time was measured by a validated ELISA. The related sex-averaged pharmacokinetic parameters were assessed using noncompartmental analysis models by WinNonlin 6.1.

# Supplementary Results

## **Design and generation of PSB205**

A recombinant antibody is typically produced by a single engineered cell line in which the heavy chain (HC) and the light chain (LC) of the antibody are co-expressed, correctly assembled, and secreted. In order to produce two antibodies together, two different HCs and LCs need to be introduced in the same host cell. Due to random pairing of the HC and the LC, many undesirable by products can be generated (Fig. 1A). We specifically made changes in the HC/HC and HC/LC interface residues in such a way that the correct assembly of cognate HC/HC and HC/LC pairing is strongly favored. When these uniquely designed HC pairing keys and LC pairing keys were introduced, the two different antibodies could be expressed together without any mispaired species. The product generated by this antibody engineering technology platform contains a mixture of two recombinant antibodies in a fixed ratio, and it is designated as a MabPair molecule. PSB205 is a MabPair product developed to target PD-1 and CTLA-4, two key immune checkpoint regulators.

PSB103 (anti-PD-1 IgG4) and PSB105 (anti-CTLA-4 IgG1) were produced together in the CHO cell line at a fixed ratio of 2:1 (Fig. 1B). The relative ratio of anti-PD-1 to anti-CTLA-4 antibodies in PSB205 was determined by using allometrically scaled PK simulations for its components. The simulation predicted that when PSB205 is dosed at three-week intervals, it will achieve a different level of steady-state exposure for the anti-PD-1 and anti-CTLA-4 antibodies (data not shown). A single mutation at arginine 255 was introduced in the Fc region of PSB105 to reduce the binding to FcRn (Supplementary Table 1), leading to a faster clearance and shortening of the antibody t_1/2_ *in vivo* for anti-CTLA-4 compared to ipilimumab (t1/2: 109 vs. 397 h; clearance: 0.489 vs. 0.0948 mL/h/kg; Supplementary Table 2). PSB205 was manufactured as a single product, and its purity and product quality were fully characterized by using a panel of analytical methods. No detectable mispairing species was found in the product (Fig. 1C–E).

## **Block binding ability of PSB205**

The ability of PSB205 and its anti-PD-1 component (PSB103) or anti-CTLA-4 component (PSB105) to block the PD-1:programmed death-ligand 1 (PD-L1) interaction or CTLA-4:B7-1/B7-2 interaction was evaluated using two different dual-cell reporter assays. As displayed in Supplementary Fig. 1, both PSB103 and PSB205 mediated concentration-dependent inhibition of the PD-L1:PD-1 interaction that enabled activation of nuclear factor of activated T cells and an increased luciferase signal. The results showed that the EC50 values for PSB103 and PSB205 were 1.6 nM and 1.7 nM, respectively; PSB105 and PSB205 also released CTLA-4-mediated inhibition in the reporter assay, with EC50 values for PSB105 and PSB205 of 3.78 nM and 4.12 nM, respectively.

## **Functional assessment of PSB205**

Dendritic cells (DCs) express costimulatory (B7-1 and B7-2) and coinhibitory (PD-L1) molecules to engage CD28/CTLA-4 and PD-1 expressed by T cells, respectively. To determine how PSB205 affects T-cell stimulation by DCs, immature DCs derived from the monocytes of an alloreactive donor were used to stimulate purified T cells. Both PSB103 and PSB205 increased interferon-gamma (IFN-γ) production by T cells at different DC/T cell ratios, while anti-CTLA-4 alone had minimal effects on IFN-γ production (Supplementary Fig. 2).

In addition, the combination of PSB103 and PSB105 at a ratio of 2:1 in PSB205 can achieve a synergistic effect at stimulating CMV-specific CD8^+^ T cells. Cytomegalovirus (CMV) lysate from CMV-infected cells was used to stimulate PBMCs from a seropositive individual. We enumerated the expansion of CD8^+^ T cells specific for pp65 of CMV on day 7 using a dextramer from Immudex: HLA-A*0201/NLVPMVATV. As shown in Supplementary Fig. 3, higher percentages and absolute numbers of CMV^+^ CD8^+^ T cells were recovered from the PSB205-treated group than the group that had been treated with either PSB103 (anti-PD-1) or PSB105 (anti-CTLA-4) alone.

## **PSB205 exhibits antitumor activity in humanized animal tumor models**

Next, we tested the efficacy of PSB205 in animal models. Previous studies have shown that the combination of anti-mouse PD-1 and anti-mouse CTLA-4 antibodies demonstrates a synergistic effect in controlling tumor growth in various murine models.^14^ However, due to the lack of cross-reactivity, these models are not suitable to test PSB103, PSB105, and PSB205. To this end, we used an engineered NOD-Prkdcscid IL-2Rγ-null (NCG) mouse strain that lacks T cells, B cells, and natural killer cells. Human PBMCs were grafted onto these mice without rejection. When tumor cells of human origin (i.e., HCC827a) were implanted into NCG/human PBMC mice, the human T cells mounted a successful immune reaction to the tumor. As shown in Supplementary Fig. 4A, the combination of PSB103 and PSB105 at a 2:1 ratio (PSB205) effectively controlled HCC827 tumor growth, whereas either PSB103 or PSB105 alone did not show any effect in this model. We further tested PSB205 in the Jeko-1 tumor model, where the tumor grows faster than HCC827. As shown in Supplementary Fig. 4B, either PSB103 or PSB105 alone significantly inhibited Jeko-1 tumor growth, but PSB205 was more effective than PSB103 alone.

## **PK profiles of PSB205 in animal models**

The PK profiles of PSB103 and PSB105 were evaluated individually in single-dose exploratory experiments in cynomolgus monkeys. Systemic exposure was achieved in all animals following a single intravenous injection. The average terminal t_1/2_ was determined to be 297 h for PSB103 (Supplementary Table 2). PSB105 showed an increased rate of clearance and reduced systemic exposure compared to those of ipilimumab. The t_1/2_ values of PSB105 and ipilimumab were 109 h and 397 h, respectively (Supplementary Table 2). These findings are at least partially attributed to the reduced FcRn affinity in PSB105.

**Supplementary Figure legends**

**Supplementary Figure 1. PSB205 and PSB103 Inhibited PD-1 Binding and Functional Activity (left) and PSB205 and PSB105 Inhibited CTLA4-mediated Inhibitory Activities (right) in Dual-cell Reporter Assays.**


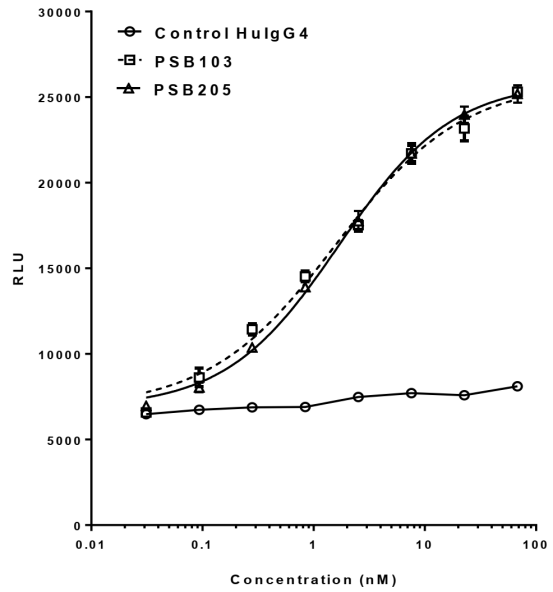

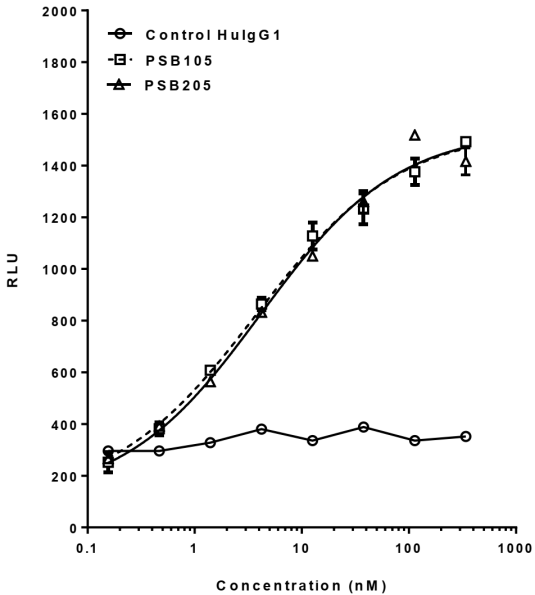


**Supplementary Figure 2. Enhanced T-cell Activation in Allo-MLR**

IFN-g=Interferon gamma; MLR=mixed lymphocyte reaction; 20F5=PSB205


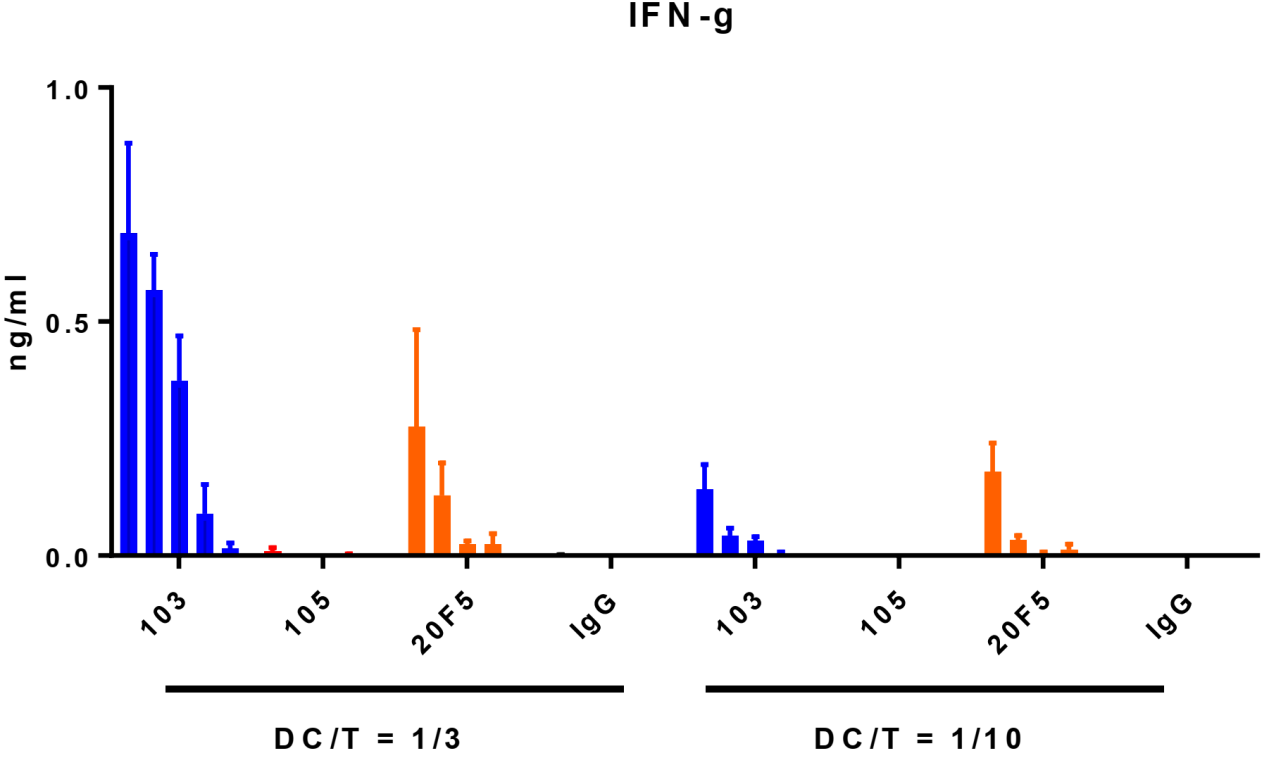


**Supplementary Figure 3.** PBMCs from a HLA-CMV pp65-positive donor were stimulated with CMV (3 μg/mL) lysate for 7 days in the presence of various antibodies in duplicate: IgG1 (5 μg/mL), PSB103 (5 μg/mL), PSB105 (2.5 μg/mL), and PSB205 (5 μg/mL). The numbers of CMVpp65-positive CD8 T cells in the culture were enumerated by flow cytometry.


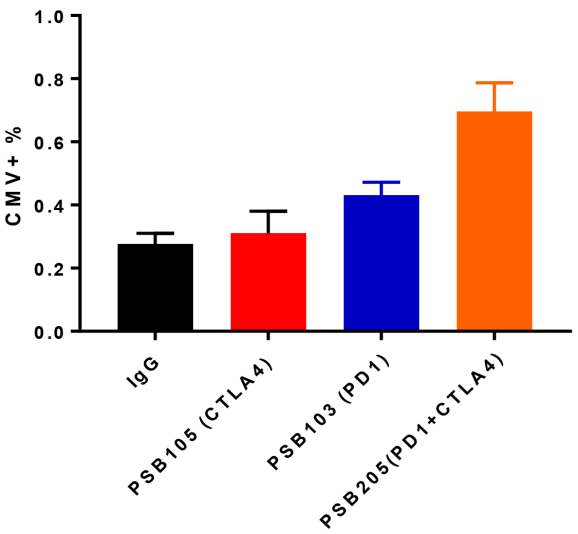

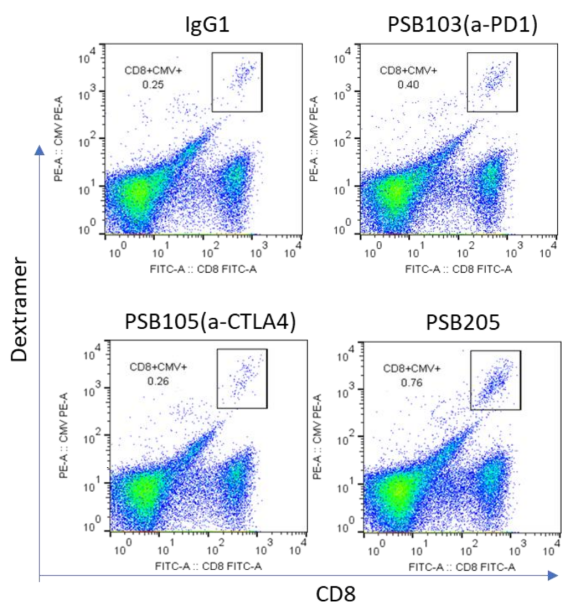


**Supplementary Figure 4. *In-vivo* assay. (A)** HCC827 cells were implanted on NOD-Prkdcscid IL-2Rγ-null (NCG) mice. When the tumor sizes reached 60–80 mm^3^, human PBMCs from a healthy donor was used to reconstitute NCG mice, as described in the Materials and Methods section. Control human IgG1 (7.5 mg/kg, *n*=5), PSB103 (5 mg/kg, *n*=5), PSB105 (2.5 mg/kg, *n*=5), and PSB103 mixed with PSB105 at a 2:1 ratio (7.5 mg/kg, *n*=5) were intraperitoneally injected twice a week for 3 weeks. **(B)** Jeko-1 were implanted on NCG mice. When the tumor sizes reached 80–100 mm^3^, human PBMCs from a healthy donor were used to reconstitute NCG mice as described in the Materials and Methods section. Control human IgG1 (7.5 mg/kg, *n*=5), PSB103 (5 mg/kg, *n*=5), PSB105 (2.5 mg/kg, *n*=5), and PSB103 mixed with PSB105 at a 2:1 ratio (7.5 mg/kg, *n*=5) were i.p. injected twice a week for 3 weeks.

**A**


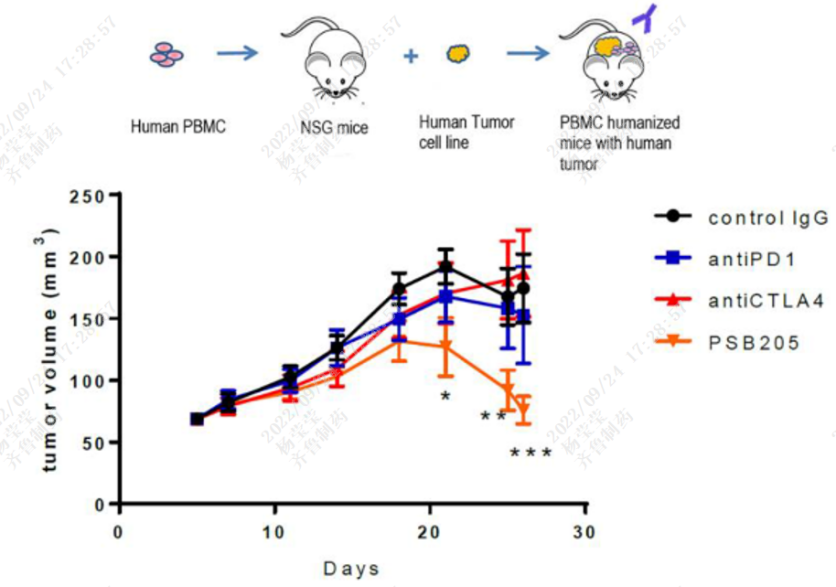


**B**


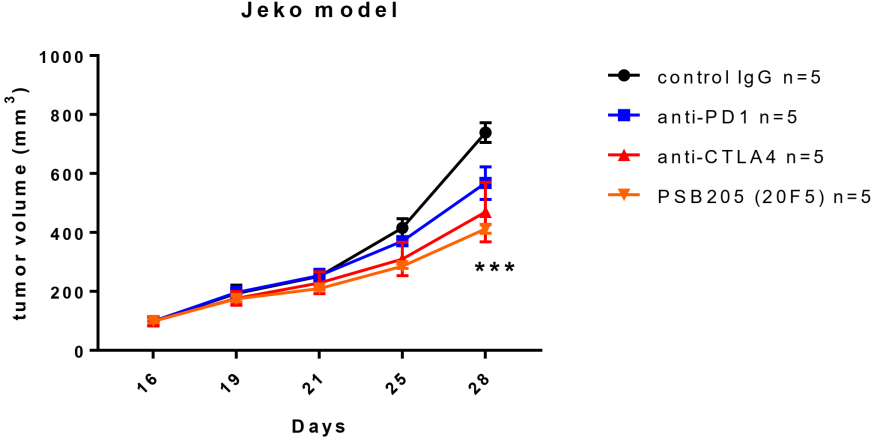


**Supplementary Figure 5.** Impact of different covariates on anti-PD-1 **(A)** and anti-CTLA-4 **(B)** steady-state exposure. The C_max,ss_, C_trough,ss_, and AUC_ss_ of 5 mg/kg Q3W in subjects with different sexes, ADA states, baseline body weight (BBWT), albumin concentration (ALB), and tumor burden (BTB) compared with typical individuals. The points in the figure represent the median steady-state exposure ratio of different patients to typical subjects, and the error bars represent 90% confidence intervals for the ratio.

**A**


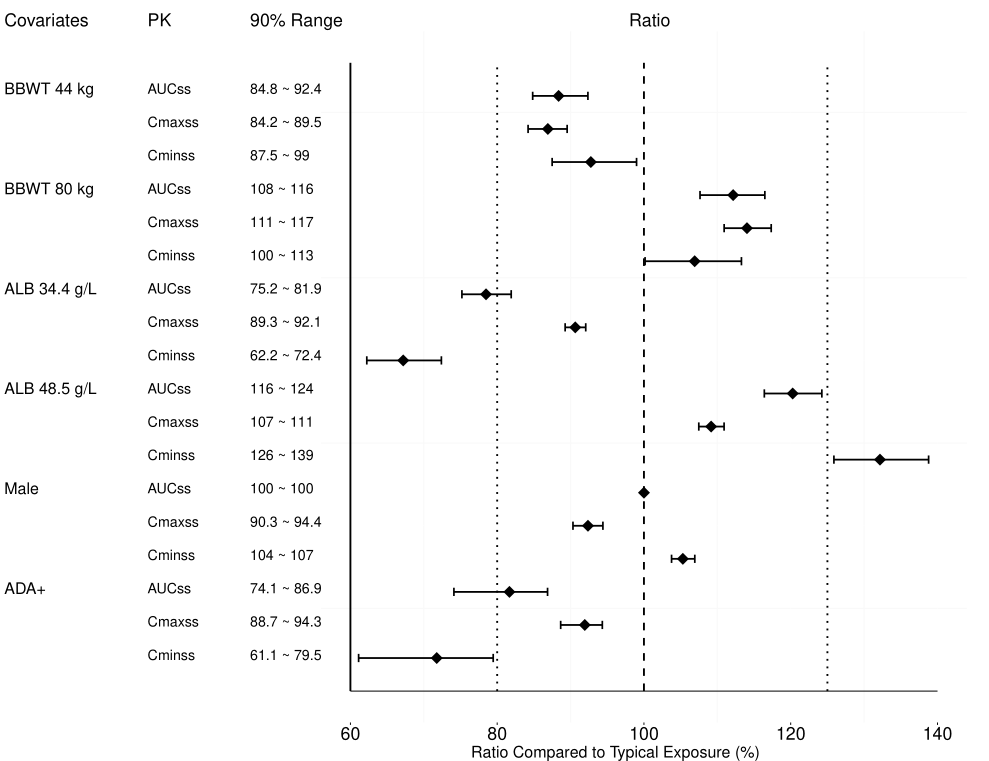


**B**


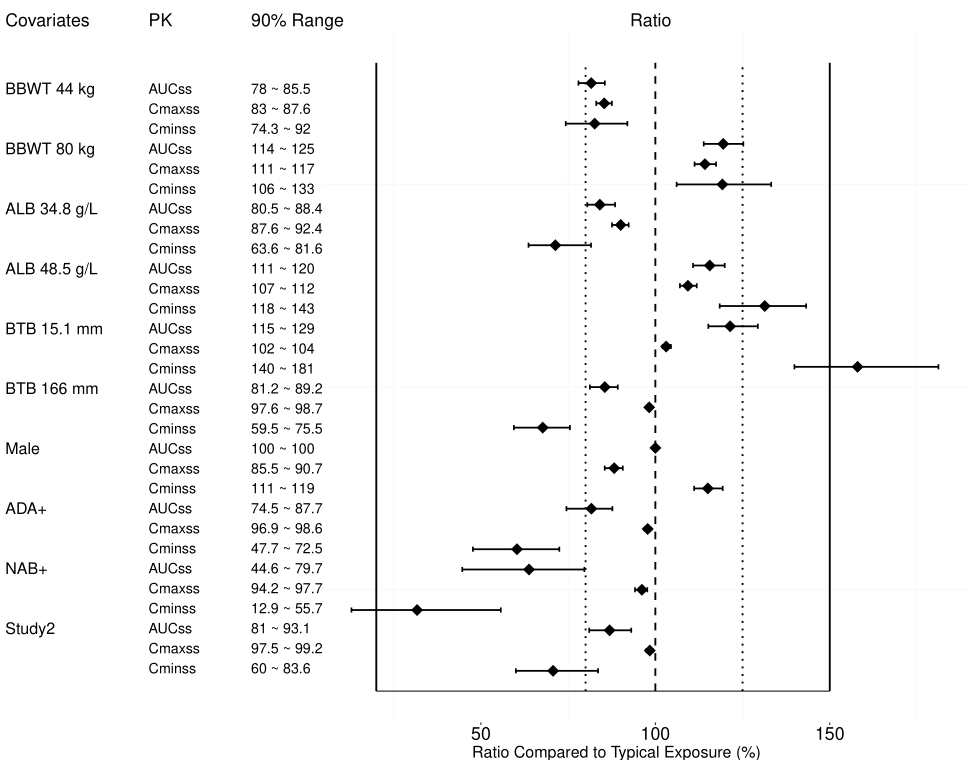


**Supplementary Figure 6.** The Kaplan–Meier curves for the DoR of all patients receiving QL1706 at RP2D (A), for immunotherapy-naive NSCLC (B), NPC (C), and CC (D).

**
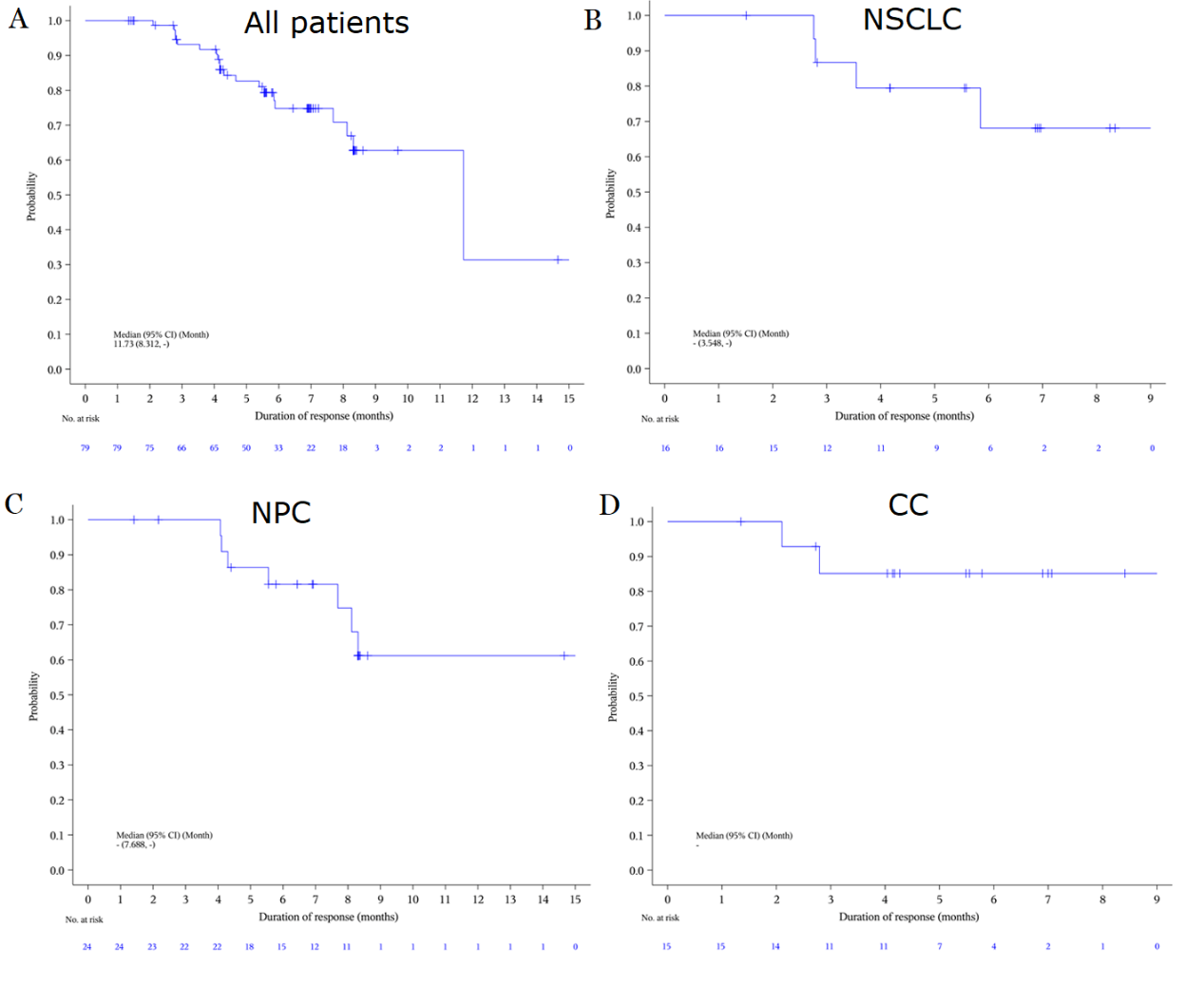
**

**Supplementary Table 1.** **Summary of anti-CTLA-4 IgG1 variants binding to human FcRn/β 2M complex at pH 6.0 by Biacore analysis.**

| **Anti-CTLA4 clone** | **Mutation** | **Concentration (nM)** | **Rmax (RU) at pH 6.0** | **Relative FcRn Binding** |
| --- | --- | --- | --- | --- |
| 11F4 (PSB105P) | Parent | 125 | 132 | 1.00 |
|  |  | 62.5 | 85.1 | 1.00 |
| 11F4 (PSB105) | R255K | 125 | 101 | 0.77 |
|  |  | 62.5 | 73.9 | 0.87 |

**Supplementary Table 2. Sex-averaged Pharmacokinetic Parameters of Test Articles Following Single i.v. Administration in Cynomolgus Monkeys**

| Test Article | Parameter | t_1/2_  (h) | T_max_  (h) | C_max_  (μg/mL) | AUC_0-last_  (h*μg/mL) | AUC_0-∞_  (h*μg/mL) | Vz  (mL/kg) | Cl  (mL/h/kg) |
| --- | --- | --- | --- | --- | --- | --- | --- | --- |
| PSB103 (29102) | N^a^ | 4 | 4 | 4 | 4 | 4 | 4 | 4 |
|  | Mean | 297 | 2.17^b^ | 204 | 37300 | 48800 | 43.9 | 0.106 |
|  | SD | 64.8 | 3.89 | 23.5 | 3540 | 10800 | 1.05 | 0.0203 |
| Human IgG4 (16102) | N^a^ | 4 | 4 | 4 | 4 | 4 | 4 | 4 |
|  | Mean | 181 | 2.06 ^b^ | 138 | 16900 | 21000 | 64.5 | 0.270 |
|  | SD | 51.0 | 3.96 | 19.9 | 8060 | 8420 | 9.27 | 0.108 |
| PSB105 (10511) | N | 2 | 2 | 2 | 2 | 2 | 2 | 2 |
|  | Mean | 109 | 0.290 | 116 | 6080 | 6140 | 76.6 | 0.489 |
|  | SD | 1.66 | 0.297 | 26.1 | 283 | 287 | 4.75 | 0.0229 |
| PSB105P (10511P) | N | 2 | 2 | 2 | 2 | 2 | 2 | 2 |
|  | Mean | 125 | 0.08 | 153 | 10100 | 10300 | 52.3 | 0.291 |
|  | SD | 21.2 | 0.00 | 4.67 | 440 | 631 | 5.69 | 0.0177 |
| ipilimumab | N | 2 | 2 | 2 | 2 | 2 | 2 | 2 |
|  | Mean | 397 | 0.08 | 128 | 22500 | 31900 | 53.5 | 0.0948 |
|  | SD | 104 | 0.00 | 4.67 | 93.1 | 3740 | 7.80 | 0.0111 |

Note: a: aliquots of serum; b: CV>100

**Supplementary Table 3. Treatment-emergent adverse events in all treated patients**

|  | **Phase I (*n*=99)** | | | **Phase Ib (*n*=419)** | | **Total (*n*=518)** | | | |
| --- | --- | --- | --- | --- | --- | --- | --- | --- | --- |
|  | **Any Grade** | | **Grade≥3** | **Any Grade** | **Grade≥3** | | **Any Grade** | **Grade≥3** | |
| TEAE | 98 (99.0) | 28 (28.3) | | 373 (89.0) | 125 (29.8) | | 471 (90.9) | | 153 (29.5) |
| Severe TEAE | 21 (21.2) | 18 (18.2) | | 109 (26.0) | 83 (19.8) | | 130 (25.1) | | 101 (19.5) |
| TEAE leading to dose interruption | 10 (10.1) | 6 (6.1) | | 91 (21.7) | 54 (12.9) | | 101 (19.5) | | 60 (11.6) |
| TEAE leading to drop out | 11 (11.1) | 11 (11.1) | | 31 ( 7.4) | 25 (6.0) | | 42 (8.1) | | 36 (6.9) |
| TEAE leading to death | 2 (2.0) | 2 (2.0) | | 28 (6.7) | 28 (6.7) | | 30 (5.8) | | 30 (5.8) |
| **TEAE occurring in ≥10% patients** | | | | | | | | | |
| Anemia | 54 (54.5) | 7 (7.1) | | 114 (27.2) | 15 ( 3.6) | | 168 (32.4) | | 22 (4.2) |
| Hypoalbuminemia | 43 (43.4) | 0 | | 69 (16.5) | 0 | | 112 (21.6) | | 0 |
| Rash | 34 (34.3) | 1 (1.0) | | 71 (16.9) | 2 (0.5) | | 105 (20.3) | | 3 (0.6) |
| Hyponatremia | 29 (29.3) | 0 | | 55 (13.1) | 1 ( 0.2) | | 84 (16.2) | | 1 (0.2) |
| AST increase | 29 (29.3) | 3 (3.0) | | 52 (12.4) | 4 (1.0) | | 81 (15.6) | | 7 (1.4) |
| Hypothyroidism | 23 (23.2) | 1 (1.0) | | 52 (12.4) | 0 | | 75 (14.5) | | 1 (0.2) |
| Appetite decrease | 16 (16.2) | 0 | | 56 (13.4) | 5 (1.2) | | 72 (13.9) | | 5 (1.0) |
| Fatigue | 17 (17.2) | 0 | | 55 (13.1) | 3 ( 0.7) | | 72 (13.9) | | 3 (0.6) |
| Pruritus | 33 (33.3) | 0 | | 38 (9.1) | 0 | | 71 (13.7) | | 0 |
| Pyrexia | 13 (13.1) | 0 | | 57 (13.6) | 0 | | 70 (13.5) | | 0 |
| ALT increase | 20 (20.2) | 2 (2.0) | | 46 (11.0) | 4 (1.0) | | 66 (12.7) | | 6 (1.2) |
| LDL increase | 37 (37.4) | 0 | | 22 (5.3) | 0 | | 59 (11.4) | | 0 |
| Weight loss | 13 (13.1) | 0 | | 45 (10.7) | 0 | | 58 (11.2) | | 0 |
| Hyperthyroidism | 14 (14.1) | 0 | | 40 ( 9.5) | 0 | | 54 (10.4) | | 0 |
| Lipase increase | 18 (18.2) | 1 (1.0) | | 34 (8.1) | 3 (0.7) | | 52 (10.0) | | 4 (0.8) |

Abbreviations: TEAE, treatment-emergent adverse event; AST, aspartate aminotransferase; ALT, alanine aminotransferase; LDL, low-density lipoprotein. Supplementary Table 4. Response by PD-1 expression status in patients receiving QL1706 at RP2D with PD-1 data.

|  | **All patients**  **(*n*=112)** | | **Primary tumor type** | | | | | | |
| --- | --- | --- | --- | --- | --- | --- | --- | --- | --- |
|  |  |  | **NSCLC (*n*=25)** | | **NPC (*n*=26)** | | **Cervical cancer (*n*=30)** | | |
|  | **CPS＜1**  **(*n*=36)** | **CPS≥1**  **(*n*=76)** | **CPS＜1**  **(*n*=8)** | **CPS≥1**  **(*n*=17)** | **CPS<1**  **(*n*=2)** | **CPS≥1**  **(*n*=24)** | | **CPS<1**  **(*n*=8)** | **CPS≥1**  **(*n*=22)** |
| CR | 0 | 1 (1.3) | 0 | 0 | 0 | 0 | | 0 | 1 (4.5) |
| PR | 6 (16.7) | 20 (26.3) | 1 (12.5) | 5 (29.4) | 1 (50.0) | 4 (16.7) | | 2 (25.0) | 7 (31.8) |
| SD | 8 (22.2) | 21 (27.6) | 2 (25.0) | 7 (41.2) | 0 | 5 (20.8) | | 2 (25.0) | 6 (27.3) |
| PD | 17 (47.3) | 29 (38.2) | 5 (62.5) | 5 (29.4) | 1 (50.0) | 13 (54.2) | | 2 (25.0) | 6 (27.3) |
| ND | 5 (13.9) | 5 (6.6) | 0 | 0 | 0 | 2 (8.3) | | 2 (25.0) | 2 (9.1) |
| Confirmed ORR, *n* (%, 95% CI) | 6 (16.7)  (6.4, 32.9) | 21 (27.6)  (18.0, 39.1) | 1 (12.5)  (0.3, 52.7) | 5 (29.4)  (10.3, 56.0) | 1 (50.0)  (1.3, 98.7) | 4 (16.7)  (4.8, 37.4) | | 2 (25.0)  (3.2, 65.1) | 8 (36.4)  (17.28, 59.3) |
| DCR, *n* (%, 95% CI) | 14 (38.9)  (23.2, 56.5) | 41 (53.9)  (42.1, 65.5) | 3 (37.5)  (8.5, 75.5) | 12 (70.6)  (44.0, 89.7) | 1 (50.0)  (1.3, 98.7) | 9 (37.5)  (18.8, 59.4) | | 4 (50.0,  (15.7, 84.2) | 14 (63.6)  (40.7, 82.8) |

Abbreviations: NSCLC, non-small-cell lung cancer; NPC, nasopharyngeal carcinoma; CC, cervical cancer; CR, complete response; PR, partial response; SD, stable disease; PD, progressive disease; ND, not determined; ORR, objective response rate; DCR, disease control rate; CI, confidence interval.

Supplementary Table 5. Pharmacokinetic parameters of anti-CTLA-4 and anti-PD-1 after intravenous infusion of QL1706 at cycle 1 and cycle 6 in phase I.

| **PK Parameter**  **(unit)** |  | | | **Cycle 1 (First-dose)** | | | | | **Cycle 6 (Multiple-dose)** | | |  |  |
| --- | --- | --- | --- | --- | --- | --- | --- | --- | --- | --- | --- | --- | --- |
|  | **0.3 mg/kg**  **(*n*=1)** | | **1 mg/kg**  **(*n*=6)** | | **3 mg/kg**  **(*n*=6)** | **5 mg/kg**  **(*n*=49)** | **7.5 mg/kg (*n*=31)** | **10 mg/kg**  **(*n*=6)** | **1 mg/kg**  **(*n*=3)** | **3 mg/kg**  **(*n*=3)** | **5 mg/kg**  **(*n*=27)** | **7.5 mg/kg (*n*=17)** | **10 mg/kg**  **(*n*=2)** |
|  | | **anti-CTLA-4** | | | | | | | | | |  |  |
| **C_max_ (μg/mL)** | 2.27 | | 7.86±0.883 (11.2) | | 24.1±2.54  (10.5) | 38.2±5.95  (15.6) | 52.9±9.53  (18.0) | 73.6±14.2  (19.3) | 8.06±1.32  (16.4) | 23.9±0.525  (2.2) | 37.2±8.22  (22.1) | 64.9±16.7  (25.8) | 82.4±24.1  (29.3) |
| **T_max_ (h)** | 2.50 | | 2.50  (0.50, 48.40) | | 0.54  (0.52, 2.50) | 0.55  (0.5, 8.53) | 0.533  (0.517, 48.6) | 0.625  (0.52, 2.53) | 0.52  (0.52, 0.55) | 2.5  (0.52, 24.6) | 0.53  (0.5, 72.3) | 2.50  (0.517, 8.57) | 0.53  (0.53, 0.53) |
| **AUC_0-t_ (μg·h/mL)** | 123 | | 1210±333  (27.5) | | 3110±1000  (32.3) | 5020±1720  (34.3) | 6860±2690  (39.3) | 8380±3270  (39) | 1390±40.2  (2.9) | 2940±340  (11.6) | 6540±2850  (43.6) | 8100±3450  (42.6) | 10700±7100  (66.1) |
| **T_last_ (h)** | 168 | | 503  (335, 529) | | 480  (455, 504) | 504  (167, 531) | 504  (73.7, 695) | 468  (335, 671) | 505  (504, 647) | 504  (503, 504) | 505  (8.33, 557) | 480  (71.9, 505) | 407  (335, 480) |
| **AUC_0-21d_ (μg·h/mL)** | 176 | | 1230±308  (25) | | 3120±995  (31.9) | 5130±1780  (34.6) | 6900±2600  (37.7) | 8620±3460  (40.1) | 1350±66  (4.87) | 2940±340  (11.5) | 6770±2520  (37.2) [26] | 8400±3590  (42.7) | 10900±7280  (66.8) |
| **AUC_0-∞_ (μg·h/mL)** | 185 | | 1310±343  (26.1) | | 3280±1100  (33.4) | 5510±2170  (39.5) | 7330±2920  (39.9) | 9360±4630  (49.5) | 1590±86.6 (5.43) | 3060±380  (12.4) | 7760±3420  (44) [26] | 9230±4520  (48.9) | 12400±9390  (75.8) |
| **AUC__% Extrap_ (%)** | 33.5 | | 8.18±2.11  (25.7) | | 5.19±1.28  (24.7) | 6.75±8.27  (123) | 6.17±5.55  (90.1) | 7.68±7.84  (102) | 12.8±3.95 (30.7) | 3.83±1.4  (36.6) | 10.3±7.24  (70.4) [26] | 10.8±9.49  (87.6) | 8.75±11.8  (135) |
| **CL (mL/h/kg)** | 0.571 | | 0.29±0.106  (36.5) | | 0.355±0.131  (36.9) | 0.37±0.15  (40.5) | 0.419±0.162  (38.6) | 0.43±0.135  (31.5) | 0.221±0.0118  (5.34) | 0.348±0.0428  (12.3) | 0.269±0.118  (44) [26] | 0.377±0.251  (66.5) | 0.399±0.302  (75.8) |
| **V_z_ (mL/kg)** | 98.7 | | 49.8±4.3 (28.7) | | 58.6±21.6  (36.8) | 57.2±12 (21) | 62.8±19.2 (30.6) | 65.6±11.9  (18.1) | 60.6±1.91  (3.14) | 56±8.27  (14.8) | 53±12.2  (23.1) [26] | 56.3±19.4  (34.5) | 44.9±10.4  (23.3) |
| **λ_z_ (1/h)** | 0.00578 | | 0.00576±0.00046 (7.99) | | 0.00607±0.000773 (12.7) | 0.00638±0.00176 (27.6) | 0.00684±0.00259 (37.8) | 0.00665±0.00221 (33.3) | 0.00365±0.0000865  (2.37) | 0.00628±0.000943  (15) | 0.00506±0.00165  (32.6) [26] | 0.00640±0.00234 (36.6) | 0.00993±0.00904 (91) |
| **t_1/2_ (h)** | 120 | | 121±9.52 (7.86) | | 116±14.9 (12.9) | 118±34.5 (29.3) | 112±33.1 (29.5) | 118±54.5 (46) | 190±4.49  (2.36) | 112±16.2  (14.5) | 153±55.8  (36.4) [26] | 121±39.9  (33.1) | 119±109  (91) |
| **C_trough_ (μg/mL)** | BQL | | 0.568±.0722 (12.7) [4] | | 1.02±0.434 (42.7) | 1.91±1.61 (84.3) [42] | 2.45±1.98 (80.7) [27] | 3.51±5.34 (152) [4] | 0.749±0.246  (32.8) | 0.718±0.236  (32.9) | 3.51±2.71  (77.2) [25] | 4.40±4.56  (104) [12] | 11.5 [1] |
| **R_ac__C_max_** |  | |  | |  |  |  |  | 1.041±0.061  (5.8) | 0.935±0.084  (9) | 0.984±0.218  (22.2) | 1.227±0.184  (15) | 1.11±0.052  (4.7) |
| **R_ac__AUC_0-21d_** |  | |  | |  |  |  |  | 0.964±0.142  (14.7) | 1.227±0.275  (22.4) | 1.212±0.336  (27.7) [26] | 1.151±0.381  (33.1) | 0.924±0.168  (18.2) |
| **R_ac__C_trough_** |  | |  | |  |  |  |  | 1.33±0.575  (43.4) | 0.97±0.189  (19.5) | 1.78±1.27  (71.3) [23] | 1.85±1.32  (71.6) [10] | 1 [1] |
|  | | **anti-PD-1** | | | | | | | | | |  |  |
| **C_max_ (μg/mL)** | 4.25 | | 13.8±2.65 (19.2) | | 49.5±5.8  (11.7) | 76.6±13.1  (17.1) | 112±20.1  (17.9) | 157±23.4  (14.9) | 21.2±2.59 (12.2) | 68.7±3.61  (5.26) | 109±23.6  (21.6) | 144±33.0  (22.9) | 212±85.8  (40.4) |
| **T_max_ (h)** | 2.5 | | 0.52  (0.5, 24.6) | | 0.53  (0.52, 0.57) | 0.55  (0.50, 167) | 0.55  (0.52, 8.5) | 1.58  (0.52, 8.5) | 2.5  (0.52, 2.5) | 0.52  (0.52, 0.53) | 2.48  (0.5, 24.3) | 2.53  (0.517, 24.1) | 1.52  (0.53,2.5) |
| **AUC_0-t_ (μg·h/mL)** | 651 | | 3200±532  (16.6) | | 9690±2050  (21.2) | 15700±3190  (20.3) | 20800±5410  (26.1) | 28600±4530  (15.9) | 5810±308  (5.31) | 17300±740  (4.27) | 30500±7180  (23.6) | 41800±27500  (65.8) | 55900±19900  (35.5) |
| **T_last_ (h)** | 502 | | 504  (456, 574) | | 480  (455, 504) | 504  (167, 838) | 504  (73.7, 792) | 468  (335, 671) | 505  (504, 647) | 504  (503, 504) | 505  (335, 557) | 335  (71.6, 504) | 480  (480, 480) |
| **AUC_0-21d_ (μg·h/mL)** | 652 | | 3190±593  (18.6) | | 9900±1940  (19.6) | 15700±2800  (17.8) [48] | 20800±4420  (21.3) | 29200±4170  (14.3) | 5570±727  (13) | 17300±732  (4.22) | 30400±6510  (21.4) | 36700±11300  (30.6) | 57900±20800  (35.9) |
| **AUC_0-∞_ (μg·h/mL)** | 843 | | 4240±861  (20.3) | | 14000±3140  (22.4) | 22500±6850  (30.4) [48] | 30300±12600  (41.8) | 35900±9140  (25.5) | 10400±2000  (19.2) | 27000±553  (2.05) | 55200±17700  (32) | 67400±30100  (44.7) | 109000±57800  (53.1) |
| **AUC__% Extrap_ (%)** | 22.8 | | 24±5.94 (24.8) | | 30.7±3.96 (12.9) | 28.1±11.3  (40.4) [48] | 28.9±11.8  (40.9) | 18.6±10.7 (57.4) | 43.1±8.97  (20.8) | 35.9±1.46  (4.06) | 42.2±10.9  (25.9) | 39.1±14.8  (37.9) | 45.8±10.5  (23) |
| **CL (mL/h/kg)** | 0.23 | | 0.159±0.0349 (22) | | 0.146±0.0404 (27.7) | 0.157±0.049(31.2) [48] | 0.181±0.0616  (34.0) | 0.19±0.0435 (22.9) | 0.0639±0.0135  (21.2) | 0.072±0.00149  (2.07) | 0.0667±0.0286  (42.9) | 0.0828±0.0283  (34.1) | 0.0693±0.0368  (53.1) |
| **V_z_ (mL/kg)** | 84 | | 57.1±11.7 (20.5) | | 60±14.6 (24.3) | 59.2±11.9  (20) [48] | 68.6±14.2  (20.8) | 51.2±14.7  (28.8) | 43.3±4.45  (10.3) | 35.5±2.75  (7.76) | 37.4±9.81  (26.2) | 47.5±12.6  (26.5) | 40.2±11.5  (28.7) |
| **λ_z_ (1/h)** | 0.00275 | | 0.0028±0.000433 (15.5) | | 0.00245±0.000397 (16.2) | 0.00276±0.00103 (37.5) [48] | 0.00279±0.00141  (50.6) | 0.00399±0.00142 (35.7) | 0.00147±0.00017  (11.6) | 0.00204±0.00012  (5.91) | 0.00179±0.000546  (30.4) | 0.00175±0.000446  (25.5) | 0.00166±0.000439  (26.4) |
| **t_1/2_ (h)** | 252 | | 253±39.4 (15.6) | | 290±47.5 (16.4) | 285±114  (40) [48] | 293±122  (41.6) | 201±95.4 (47.5) | 477±55.2  (11.6) | 341±20  (5.87) | 425±137  (32.3) | 422±113  (26.8) | 433±114  (26.4) |
| **C_trough_ (μg/mL)** | 0.529 | | 2.8±0.801  (28.6) | | 10.4±2.23  (21.5) | 14.9±5.26  (35.2) [48] | 20.3±7.66  (37.7) [27] | 22.1±15.5  (70.1) [4] | 6.57±1.86  (28.3) | 19.7±0.758  (3.84) | 37.5±10.3 (27.5) [26] | 39.8±19.3  (48.6) [16] | 79.6±39.8  (50) |
| **R_ac__C_max_** |  | |  | |  |  |  |  | 1.636±0.484  (29.6) | 1.306±0.141  (10.8) | 1.453±0.255  (17.5) | 1318±0.346  (26.3) | 1.389±0.243  (17.5) |
| **R_ac__AUC_0-21d_** |  | |  | |  |  |  |  | 2.22±1.02  (45.8) | 2.23±0.761  (34.1) | 2.27±0.618  (27.2) [26] | 2.10±1.33  (63.3) [15] | 2.33±0.276  (11.9) |
| **R_ac__C_trough_** |  | |  | |  |  |  |  | 1.838±0.619  (33.7) | 1.908±0.499  (26.2) | 1.87±0.279  (14.9) | 1.724±0.463  (26.8) | 1.779±0.327  (18.4) |

Notes：T_max_ and T_last_ are presented as Median (Min, Max), and other parameters are presented as Mean ± SD (CV%)[n].

Abbreviations: PK, pharmacokinetic; C_max_, maximum concentration; T_max_, time to maximum concentration; AUC, area under time-concentration curve; CL, clearance

Supplementary Table 6. Parameters estimated from the final model for anti-PD-1 in phase I.

| Parameter | Units | Estimate | RSE (%) | IIV (%) | IIV_RSE (%);  Shrinkage (%) |
| --- | --- | --- | --- | --- | --- |
| CL | L/day | 0.221 | 2 | 25 | 4; 19 |
| VC | L | 2.65 | 2 | 19 | 4; 13 |
| CLD | L/day | 0.54 | 9 | -- | --; -- |
| V2 | L | 1.38 | 7 | 57 | 9; 58 |
| Residual CV | % | 20 | <1 | -- | --; 12 |
| CLMAX | -- | -0.168 | 14 | 23 | 5; 43 |
| ET50 | weak | 5.32 | 9 | 51 | 20; 71 |
| GAMA | -- | 7.46 | 22 | -- | --; -- |
| ALB on CL | -- | -1.24 | 8 | -- | --; -- |
| BBWT on VC | -- | 0.462 | 14 | -- | --; -- |
| ADA on CL | -- | 1.24 | 2 | -- | --; -- |
| BBWT on CL | -- | 0.591 | 15 | -- | --; -- |
| SEX on VC | -- | 1.15 | 2 | -- | --; -- |

Abbreviations: RSE, relative standard error; IIV, inter-individual variability; CL, clearance in the central compartment; VC, volume of distribution in the central compartment; CLD, clearance in the peripheral compartment; V2, volume of distribution in the peripheral compartment. Residual CV, proportional residual error; CLMAX, maximum change in clearance; ET50, time for 50% of maximum effect; GAMA, Hill coefficients in the sigmoid Emax equations; ALB, real-time albumin; BBWT, baseline body weight; ADA, anti-drug antibody.

Supplementary Table 7. Parameters estimated from the final model for anti-CTLA-4 in phase I.

| Parameter | Units | Estimate | RSE (%) | IIV (%) | IIV_RSE (%);  Shrinkage (%) |
| --- | --- | --- | --- | --- | --- |
| CL | L/day | 0.458 | 4 | 29 | 4; 20 |
| VC | L | 2.56 | 2 | 14 | 5; 31 |
| CLD | L/day | 0.132 | 6 | -- | --; -- |
| V2 | L | 1.19 | 9 | 97 | 9; 51 |
| Residual CV | % | 29 | <1 | -- | --; 8 |
| ADA on CL | -- | 1.23 | 1 | -- | --; -- |
| NAB on CL | -- | 1.58 | 1 | -- | --; -- |
| BBWT on VC | -- | 0.522 | 12 | -- | --; -- |
| ALB on CL | -- | -0.953 | 7 | -- | --; -- |
| GENDER on VC | -- | 1.16 | 2 | -- | --; -- |
| ALB on VC | -- | -0.513 | 14 | -- | --; -- |
| BTB on CL | -- | 0.146 | 16 | -- | --; -- |
| BBWT on CL | -- | 0.363 | 27 | -- | --; -- |
| STUDY on CL | -- | 1.15 | 4 | -- | --; -- |

RSE, relative standard error; IIV, inter-individual variability; CL, clearance in the central compartment; VC, volume of distribution in the central compartment; CLD, clearance in the peripheral compartment; V2, volume of distribution in the peripheral compartment; Residual CV, proportional residual error; ADA, anti-drug antibody; NAB, neutralizing antibody; BBWT, baseline body weight; ALB, real-time albumin; BTB, tumor burden; STUDY, study (QL1706-101 or QL1706-102).
